# Microcalcifications in breast cancer tissue studied by X-ray absorption, emission, scattering and diffraction

**DOI:** 10.1107/S1600576724011750

**Published:** 2025-02-01

**Authors:** Thomas Huthwelker, Camelia N. Borca, Davide Altamura, Liberato De Caro, Renzo Vanna, Fabio Corsi, Carlo Morasso, Greta Banfi, Giovanni Arpa, Oliver Bunk, Cinzia Giannini

**Affiliations:** a Paul Scherrer Institute Villigen PSI Switzerland; b Institute of Crystallography National Research Council (IC-CNR) Bari Italy; c Institute for Photonics and Nanotechnologies National Research Council (IFN-CNR) Milan Italy; d Department of Biomedical and Clinical Sciences University of Milan Milan Italy; e Breast Unit, Department of Surgery, Istituti Clinici Scientifici Maugeri IRCCS, Pavia, Italy; f Nanomedicine and Molecular Imaging Lab, Istituti Clinici Scientifici Maugeri IRCCS, Pavia, Italy; g Unit of Anatomic Pathology, Istituti Clinici Scientifici Maugeri IRCCS, Pavia, Italy; Oak Ridge National Laboratory, USA; North Carolina State University, USA

**Keywords:** breast cancer, microcalcifications, whitlockite, hy­droxy­apatite, trace elements, magnesium, wide-angle X-ray scattering, X-ray fluorescence, XANES

## Abstract

Microcalcifications (MC) are observed in various tissues and in relation to several diseases. For breast cancer, recent studies have reported differences in the nature of the MC and correlations to the degree of malignancy of the neoplasm. Here, investigations of benign, ductal carcinoma *in situ* (DCIS) and invasive ductal carcinoma (IDC) breast MC using X-ray fluorescence, X-ray absorption spectroscopy and wide-angle X-ray scattering are reported. While Mg has been observed in all MC, only for the benign MC has a rim of crystalline whitlockite been identified as a minor crystalline phase in addition to the major hy­droxy­apatite (HAP) one. MC in DCIS and IDC tissue exhibit a higher abundance of a high-crystallinity HAP phase in comparison with the less well ordered MC in the benign tissue. Moreover, the distribution of other trace elements in the MC, such as Na, S, Cl, Sr and Y, is observed. For the quantitative analysis of the elemental maps, the experimentally determined sample thickness in each pixel has been incorporated as an additional parameter in the fitting process to account for sample roughness.

## Abstract 2

Microcalcifications in breast cancer tissue have been investigated. Correlation of the occurrence of a rim of crystalline whitlockite and of a hy­droxy­apatite phase of high crystallinity with malignancy has been identified for the samples studied.

## 1. Introduction

Microcalcifications (MC) are present in several types of cancer, such as breast carcinoma (Cox *et al.*, 2012 ▸; Cross *et al.*, 2014 ▸; Naseem *et al.*, 2015 ▸), thyroid carcinoma (Consorti *et al.*, 2003 ▸; Paschke *et al.*, 2011 ▸), prostate cancer (Smolski *et al.*, 2015 ▸) and glioblastoma with oligodendroglial components (Deistung *et al.*, 2013 ▸). Sometimes they are the ‘signal’ of a neoplasm, which must be removed surgically as soon as possible, but they are not necessarily the expression of a malignant cancer process and can also be present in healthy tissues.

In 2020, via a small- and wide-angle X-ray scattering (SAXS/WAXS) scanning microscopy experiment, we studied ∼10 breast tissue biopsies, all containing MC classified according to the level of malignancy in five groups (B1 – normal tissue, B2 – benign, B3 – of uncertain malignancy, B5a – carcinoma *in situ*, B5b – invasive carcinoma). X-ray scattering data were used to validate a larger study on 473 MC detected on breast biopsy specimens from 56 patients characterized entirely by Raman mapping (Vanna *et al.*, 2020 ▸). The crystal phases suggested by Raman were confirmed by WAXS measurements, identifying hy­droxy­apatite (HAP) as the most abundant crystalline phase found in the explored areas. In addition, malignant samples exhibited an increase of crystallinity, *i.e.* long-range order of the HAP. The *c* value of the unit cell and the crystalline domain size along the [002] direction were found to increase with the malignancy level. It was hypothesized that these findings could be ascribed to the presence of Mg ions. In healthy conditions, biomineralization involves HAP with partial substitution of Ca ions. For the exact position of Mg^2+^ in the HAP lattice it is still controversial whether it occupies the Ca(I) site or the Ca(II) site in the HAP structure (Kolmas *et al.*, 2011 ▸). Additionally, Mg can be interstitially included (Bystrov *et al.*, 2023 ▸). If smaller cations, such as Mg ions, substitute Ca ions, they may lead to a unit-cell contraction compared with the stoichiometric crystal. Therefore, we concluded observing that the measured trend of the *c* parameter could be correlated to an altered metabolism of Mg ions, which changes with the malignancy level. Indeed, neoplastic cells, *i.e.* for B5 samples, are low in Mg cations (Castiglioni & Maier, 2011 ▸; Wolf *et al.*, 2009 ▸). Therefore, the Mg quantity available for MC might be reduced, accounting for the lattice expansion, compared with what is expected in benign biomineralization.

Additionally, the observed trend of the *c* parameter, which increases passing from benign to malignant MC, could also be associated with carbonate substitution in the HAP structures. The HAP chemical structure is known to be altered by substituted carbonate ions, which occupy two different sites within the HAP structure [see *e.g.* Peroos *et al.* (2006 ▸) and references therein]. The first type of substitution is termed as ‘type A’, which involves substituting a linear hydroxyl ion with the larger flat carbonate ion (CO_3_^2−^). In B-type substitutions, carbonate ions replace the larger phosphate ions in the HAP lattice. This type of substitution is regarded as the most preferred carbonate substitution as it is found in bone of a variety of living species. Both A- and B-type substitutions can occur simultaneously. For the breast tissue samples investigated in our studies, the lowering of the *c* parameter in benign tissues may be indicative of carbonate mainly substituting the hydroxyl site of HAP, *i.e.* A-type substitution, as has been observed in an X-ray diffraction study on MC (Gosling *et al.*, 2019 ▸).

Due to the presence of Mg, another crystalline phase, whitlockite (WHIT), could be expected together or instead of HAP. WHIT is a calcium phosphate mineral, also known as calcium mono­hydrogen phosphate. This colourless to white mineral can be found in small crystals or granular masses, usually in association with other phosphates, such as apatite. It is often a component of biological apatites (Liu *et al.*, 2013 ▸; Shah, 2023 ▸; Shah *et al.*, 2017 ▸), such as teeth and bones, and is also found in some meteorites (Fuchs, 1962 ▸). Frondel (1941 ▸) was the first to describe WHIT. He named it to honour Herbert Percy Whitlock, who was secretary and president of the Mineralogical Society of America and curator of Minerals and Gems at the American Museum of Natural History in New York. A revised analysis of our Raman spectroscopy and SAXS/WAXS data, in conjunction with initial findings from an X-ray fluorescence (XRF) study, led to the conclusion that WHIT is particularly prevalent in MC in healthy breast tissues (Morasso *et al.*, 2023 ▸).

To address the question about the chemical nature of the alterations of the microcalcification structure and thereby to provide insights into the driving mechanism of microcalcification formation, we complement our 2020 (Vanna *et al.*, 2020 ▸) and 2023 (Morasso *et al.*, 2023 ▸) studies focusing on Raman spectroscopy results with information not only on the chemical content of the MC but also on the chemical environment of the elements constituting them by analysing, in detail, selected breast samples obtained by screening biopsies from patients with different pathological situations. The first sample, benign, was obtained from a tissue sample not presenting any sign of a malignancy; the second sample, ductal carcinoma *in situ* (DCIS), was obtained from a sample characterized by the presence of initial and still localized breast cancer; and the third sample was obtained from a patient with a more aggressive breast cancer, invasive ductal carcinoma (IDC). We present a concise summary of previously published WAXS data analysis (Vanna *et al.*, 2020 ▸; Morasso *et al.*, 2023 ▸) results with the findings of our more comprehensive XRF and X-ray absorption near-edge structure (XANES) data analysis. This allows us to provide a comprehensive overview of the evidence derived from X-ray-based techniques, which can be used to investigate the occurrence of Mg or carbonate substitutions in the breast tissue samples at different disease stages. This article also presents a more detailed data analysis to further investigate the reported elemental distributions, including Mg, presented by Morasso *et al.* (2023 ▸), which were based on XRF data recorded with an incident X-ray energy of 2.5 keV. Including XRF data taken at 2.1 and 4.2 keV into the analysis, we quantify the presence of other trace elements in the MC and discuss potential pitfalls of the quantitative XRF analysis.

## 2. Experimental

### 2.1. Sample preparation

In this study, three subjects participating in the mammography screening population, affected by breast MC (Bi-Rads: 2–5) and who underwent core biopsy, were recruited. Here, we utilized parts of samples analysed in previous reports using similar sample-preparation protocols (Vanna *et al.*, 2020 ▸; Morasso *et al.*, 2023 ▸). All subjects involved were treated at the Breast Unit of Istituti Clinici Scientifici Maugeri IRCCS (Via Maugeri 4, 27100 Pavia, Lombardia, Italy) between 2019 and 2021. The study was authorized by the Ethical Committee of the institution (protocol 2281/2018 CE) and was performed in compliance with the declaration of Helsinki. According to the B categories defined by the UK National Health Service breast screening programme (Ellis *et al.*, 2004 ▸), the biopsies include one normal tissue (B1), one DCIS (B5a) and one IDC (B5b). Tissue slices were obtained from formalin-fixed paraffin-embedded tissue blocks. For each biopsy, three contiguous slices were prepared: one 4 µm slice was stained with hematoxylin and eosin for histological evaluation, one 50 µm slice was used for the WAXS analysis and one 5 µm slice was used for the XRF analysis. For WAXS analysis, the slice was mounted on top of a 0.170 mm glass coverslip and was deparaffinized following a simple protocol optimized starting from a previously reported method (Faoláin *et al.*, 2005 ▸; Vanna *et al.*, 2020 ▸). Briefly, tissue slices were immersed in two baths of hexane 95% (Merck KGaA, Darmstadt, Germany), two baths of ethanol 100% and a final bath of ethanol 95%. The procedure was repeated three times. Finally, the slices were air dried for two hours and stored in the dark before use. For XRF analysis, 5 µm slices were mounted on 1.5 µm thick Mylar foil, without further processing.

### 2.2. SAXS/WAXS microscopy

WAXS experiments were conducted at the cSAXS beamline (Bunk *et al.*, 2009 ▸) of the Swiss Light Source (SLS), Paul Scherrer Institut, Villigen, Switzerland, using similar conditions to previous experiments described by Giannini *et al.* (2014 ▸, 2019 ▸, 2021 ▸), Vanna *et al.* (2020 ▸) and Morasso *et al.* (2023 ▸). A monochromatic X-ray beam with a wavelength of 0.09117 nm corresponding to an energy of 13.6 keV was focused to 20 µm vertically and 40 µm horizontally. Eleven tissue sections were placed perpendicular to the beam and sub-areas were scanned through the beam. A Pilatus 2M pixelated area detector, placed at a sample–detector distance of 7089 mm in SAXS and 308.3 mm in WAXS, measured the scattered intensity over the exposure time and across the sample thickness. Measurements were done in WAXS geometries with step sizes of 40 µm and exposure times of 0.3 s. Data were recorded in a continuous line-scan mode along the vertical direction. The range of the momentum transfer covered by the detector was 0.02–1.8 nm^−1^ in SAXS and 0.55–36 nm^−1^ in WAXS. The transmitted X-ray beam was blocked at the detector by a beam stop. WAXS data were calibrated by LaB_6_ samples and integrated in 16 azimuthal segments.

### 2.3. XRF and XANES

The XRF and XANES spectra were taken at the PHOENIX beamline, which is located at the SLS. The source of the beamline is an elliptical undulator that delivers horizontally polarized X-ray photons. The photons were monochromated using a double-crystal monochromator employing a pair of Si(111) crystals. Contributions of higher harmonics were removed using reflections on two planar mirrors, set to an appropriate scattering angle. A Kirkpatrick–Baez system using two elliptically bent mirrors focused the photons to the desired spot size of ∼20–30 µm, which is comparable to the beamsize used in WAXS. The intensity of the incoming beam, *I*_0_, was taken from the total electron yield (TEY) of Ni-coated Mylar foil of 0.5 µm thickness. The samples were kept in the endstation at a pressure of ∼10^−6^ mbar. The fluorescent photons were detected with a four-element silicon drift detector (manufacturer: Hitachi) with an energy resolution of 150 eV.

The samples were mounted on a sample holder equipped with a rotation stage and two stepper motors. The samples were oriented relative to the detector at ∼6° to take XRF data and at 45° for taking XANES spectra. With respect to the incident X-ray beam, the samples are at 84° for XRF and 45° for XANES. Due to the finite detector opening, the effective scattering angle was roughly between 6 and 10° for the XRF data. To image the elemental composition, the sample was scanned in two dimensions, and, for each position, both an XRF spectrum and the transmitted intensity were recorded using a photodiode (Hamamatsu). For the quantitative analysis of the XRF data, only one of the four detectors was evaluated to ensure that detector geometry is well defined. For the XANES spectroscopy, all four elements were averaged to maximize the overall count rate. The exposure times were 6 s per pixel for XRF maps and 2 s per energy step for XANES.

## 3. Methods

### 3.1. Wide-angle X-ray scattering data

From the study of the angular distribution of the radiation scattered and diffracted at large angles, namely WAXS data, it is possible to identify and quantify the crystal’s sub-molecular structures, as well as microstructural information related to crystallite morphology. To analyse the information of the collected 2D WAXS data, a process of centering, calibration and azimuthal integration into a 1D WAXS profile (or a series of 16 profiles integrated over 1/16th of the angular range to capture azimuthal orientation effects) is applied for each dataset.

Each raster-scanning WAXS dataset is composed of several tens of thousands of profiles. To identify characteristic features, the 1D azimuthally averaged WAXS patterns are statistically analysed by means of a signal-classification method (Lutz-Bueno *et al.*, 2018 ▸) to extract the least correlated profiles. For the WAXS spectra shown in this article, data of all samples have been classified together, thus ensuring consistency across samples. The *q* range from 5.75 to 34.4 nm^−1^ has been used for the classification, *i.e.* the analysis is based on the central range of WAXS scattering angles. Five representative intensity profiles have been determined in this way, and for each point measured in the explored areas of the samples, the most abundant components have been determined. These five intensity profiles have also been quantitatively analysed using a Rietveld analysis (see below).

In an earlier analysis (Vanna *et al.*, 2020 ▸) where the same classification method was used for screening the data, the WHIT profile was not detected. This is due to the analysis parameters that were set for the detection of major components in terms of signal strength and peak width, whereas, in the current analysis, parameters have been tailored more towards the detection of characteristic signals that are low in intensity and narrow in peak width, *i.e.* exhibit less integrated intensity in the characteristic peaks. This different weighting is controlled by two parameters. (1) The range in *q* that is used in the classification analysis. In the present analysis, the high-intensity low-*q* data with some SAXS characteristics that can dominate the analysis have been excluded from the WAXS analysis. (2) The smoothing of the data via linear interpolation. This is used to prevent the algorithm from picking up on minor features like statistical intensity fluctuations. The smoothing window has been reduced from ten to four pixels. As a result, the WHIT peaks of comparatively low intensity and narrow peak width were detected with the parameter set described above. Details of the method and its implementation are given by Lutz-Bueno *et al.* (2018 ▸).

#### 3.1.1. Rietveld analysis

One-dimensional WAXS profiles were analysed using a Rietveld-based quantitative approach. First, the crystalline phases which account for the peak positions and peak relative intensities were identified: hexagonal HAP [Ca_5_(PO_4_)_3_(OH), Inorganic Crystal Structure Database (ICSD; https://icsd.fiz-karlsruhe.de/index.xhtml) #187840 (Pogosova *et al.*, 2013 ▸)] and rhombohedral WHIT [Ca_9.5_MgO_28_P_7_, Crystallography Open Database (COD; Gražulis *et al.*, 2009 ▸) #9012137 (Schroeder *et al.*, 1977 ▸)]. For this step, the *QualX2.0* software was used (Altomare *et al.*, 2015 ▸). Then, the *FullProf* Rietveld program (Rodríguez-Carvajal, 1993 ▸) was used to fit the whole profiles, describing with spherical harmonics the inhomogeneous peak broadening of the WAXS reflections (a phenomenological model based on a modified Scherrer formula). The free parameters were cell size and peak widths. The background was interpolated and not refined. The instrumental resolution function was considered via analysis of the diffraction pattern of the LaB_6_ NIST standard recorded under the same experimental conditions.

### 3.2. X-ray fluorescence

#### 3.2.1. XRF-map description

In an XRF experiment, the sample is irradiated by X-ray photons of energy *E*_in_, which should be higher than the binding energy of an inner-shell electron, resulting in an emitted photoelectron and an inner-shell electron hole. Filling this hole with an outer-shell electron creates a fluorescent photon with an energy characteristic of the probed atom, generating a peak in the energy spectrum of the fluorescence. Hence, the fluorescence spectrum reflects the elemental composition of the sample (see Section S1.1 of the supporting information). The aim of this XRF study is to analyse the distribution of P, Ca and other light elements, most notably Mg. Mg is a trace element in a matrix consisting mostly of Ca and P, which are heavier than Mg. A sample excitation energy of 4.2 keV causes strong fluorescence peaks for Ca (*K*α = 3.69 keV) and P (*K*α = 2.01 keV) but only a weak peak for Mg (*K*α = 1.25 keV). Moreover, when measured with an energy-dispersive silicon drift diode detector, any recorded photon creates a background signal at lower energies. Hence, the presence of the dominant fluorescence peaks from matrix elements such as P and Ca will create a background signal at lower energies, which masks the signal from lighter elements like Mg. To minimize such pitfalls, the samples have been mapped at three different energies (2.1, 2.5 and 4.2 keV), as described in Section 4.2.2.

#### 3.2.2. Quantitative analysis of XRF spectra using *PyMCA*

For a quantitative analysis, an additional complication arises from the different X-ray absorption lengths for the different energies involved. In a crystal of HAP, the three chosen excitation energies have a very similar probing depth δ of ∼5 µm (for 2.1 keV, δ = 5.3 µm; for 2.5 keV, δ = 5 µm; and for 4.2 keV, δ = 5.1 µm). However, the absorption lengths of the fluorescent photons of different elements differ significantly from 1.5 µm for Mg (1.25 keV) to 18 µm for Ca (3.69 keV) (see Section S1.1). To take care of such effects, the XRF spectra were modelled using the *PyMCA* software (Solé *et al.*, 2007 ▸). This software fits the raw XRF intensity spectra to a theoretical model, which, among other parameters, considers the photon absorption length in the sample, the photon cross section for different emission lines, the sample geometry, the composition of the detector window and the sample thickness. Analysis of the XRF maps is made relative to one reference element of known amount in the sample. The model fixes the elemental composition of the reference elements to its theoretical value, as given by the sample matrix composition. Here, we adopt the theoretical composition of HAP type B crystal as an approximation for the matrix composition (see also Section S1.2). As the P mass fraction in the different types differs by only 8%, using HAP type B as a matrix will make no practical difference for the conclusion of the analysis. The mass fraction of all other elements is then calculated from the intensity of the emission lines relative to one of the heaviest excited elements in the matrix (Ca for 4.2 keV, P for 2.5 keV, O for 2.1 keV). Hence, the Mg mass fraction derived at 4.2 keV reflects the Mg/Ca ratio, while using 2.5 keV data reflects the Mg/P ratio. By this method, both the amount of trace elements and deviations from the ideal matrix composition can be quantified. Because the calculation of the mass fraction is carried out by normalizing to the most abundant excited matrix element, the calculated mass fraction becomes meaningless outside of the MC, where the assumption about the sample matrix is incorrect.

The analysis of the XRF spectra using *PyMCA* is based on the model and the physical data as implemented in *PyMCA*, using some key assumptions. First, the effective sample thickness is derived from the X-ray absorption measurements, assuming that the matrix of the sample is HAP type B, with no other trace elements affecting the X-ray absorption. Secondly, the sample is treated as a flat surface and under ideal geometry with an infinitively small detector opening. The potential impact of pitfalls due to these assumptions and experimental uncertainties (*i.e.* sample geometry) is discussed in Section S1.5.

#### 3.2.3. Invoking the sample thickness in the XRF analysis

The samples were cut to a nominal thickness of ∼5 µm for XRF and X-ray absorption spectroscopy. We measured the effective sample thickness by X-ray absorption assuming that the sample matrix is HAP type B, as detailed in Section S1.3, and found significant variations in the effective sample thickness within the MC. Such thickness variations will introduce hard-to-control errors into the quantitative analysis of the XRF spectra, because the absorption length of the fluorescent photons of the different elements under investigation is of the order of the sample thickness and hence different for each element. To reduce the impact of such errors, for each pixel, the measured effective sample thickness, which reflects the physical absorption of photons at the specific pixel, was used as the input parameter for the fitting routine. The methodology extends the default operation mode of *PyMCA* for analysis of elemental maps, which uses the same model configuration for all fitted pixels by default. Here, we embedded the fitting routines taken from the *PyMCA* source code into specially designed Python scripts.

At first glance, this procedure seems to be a complication compared with the more ‘standard’ approach to analyse thick and polished samples which are embedded in ep­oxy, a method routinely used for geological samples. However, for samples prepared this way, the true thickness of the MC in the embedding material remains unknown, rendering quantitative analysis difficult. Feeding the measured optical density expressed as effective sample thickness back into the analysis further constrains the quantitative analysis. As the X-ray absorption of the remaining embedding paraffin and the surrounding tissue is small compared with that in the MC, a higher X-ray absorption indeed indicates the presence of the MC (see Fig. S1 of the supporting information).

#### 3.2.4. Intensity versus mass-fraction maps

The elemental distribution is represented in two different ways. First, the elemental ‘intensity maps’ (in units of photons s^−1^) reflect the spatial distribution of an element. Second, ‘mass-fraction maps’ show the elemental mass fraction of the probed element relative to the theoretical sample matrix (here, HAP type B). With the sample matrix fixed to one element, mass fractions derived from measurements at 4.2 keV show the elemental distribution relative to Ca, while maps taken at 2.5 keV show the distribution relative to P. We also provide estimates for the mass fractions of Sr and Y, which were derived from XRF spectra measured at a photon energy of 2.1 keV, below the P excitation energy. For these estimates, we use the data taken at 2.1 keV and calculate the mass fractions *w*(*Z*, 2.1 keV) of *Z* = Y, Sr, Mg, assuming that the O signal originates solely from the HAP type B matrix. As the sample may contain O from many different sources, this assumption is not fully correct, but it provides the ratio between the mass fractions of Y, Sr and Mg. Using the mass fraction *w*(Mg, 2.5 keV), as derived from the Mg/P ratio taken at 2.5 keV, we can estimate the mass fractions *w* of Sr and Y by rescaling *w*(*Z*) = *w*(*Z*, 2.1 keV) × *w*(Mg, 2.5 keV)/*w*(Mg, 2.1 keV) with *Z* = Sr, Y.

As demonstrated in Section 4.2.2, the visibility of light elements depends on the excitation energy. When fitting the XRF spectra, we sometimes did not fit all peaks. For example, the peaks for Sr and Y were omitted from the fits to spectra at 2.5 and 4.2 keV (see Figs. 2 and 3), as there is no structure in the spectra at these excitation energies. This approach has no effect on the estimated distribution of P, Mg and Ca, as the lines from these elements are either well separated from the lines of Sr and Y (Ca and Mg) or dominant (P).

#### 3.2.5. Grouping XRF spectra into ‘bins’ of similar effective sample thickness

Modelling of XRF spectra will suffer from systematic errors, such as the incomplete knowledge of the matrix composition, photon cross sections and, most notably, uncertainties of the measurement geometry, such as the finite detector opening and surface roughness. These uncertainties will cause an unknown systematic error in the treatment of the sample thickness. To reduce the impact of such systematic errors, we can group the XRF spectra by regions of similar effective thickness, average these spectra and fit them using the *PyMCA* code. Because the systematic error induced by different sample thicknesses will be similar for data in the same size bins, differences between different samples can be compared semi-quantitatively. This analysis uses the effective thickness derived from the X-ray absorption at 2.5 keV. For further detailed discussion about the limits and potential errors in the quantitative analysis see Section S1.5.

#### 3.2.6. XANES spectra

An XANES spectrum is the X-ray absorption as a function of the photon energy of the exciting photons. For the XANES measurements, the samples supported on Mylar foils were mounted at 45° relative to the incoming X-ray beam. The XANES spectra for the reference samples (CaCO_3_, amorphous CaCO_3_, aragonite, vaterite, WHIT, HAP, HAP type A and type B) were measured using both TEY and total fluorescence detection (TFY), while the samples were measured simultaneously in TFY and transmission. The raw spectra were normalized first to the incident photon flux *I*_0_. Then the pre-edge intensity was subtracted and the edge step was normalized to 1 using the standard procedures as implemented in the *Athena* software package (Ravel & Newville, 2005 ▸).

## 4. Results and discussion

### 4.1. SAXS/WAXS data

**Figure 1:**
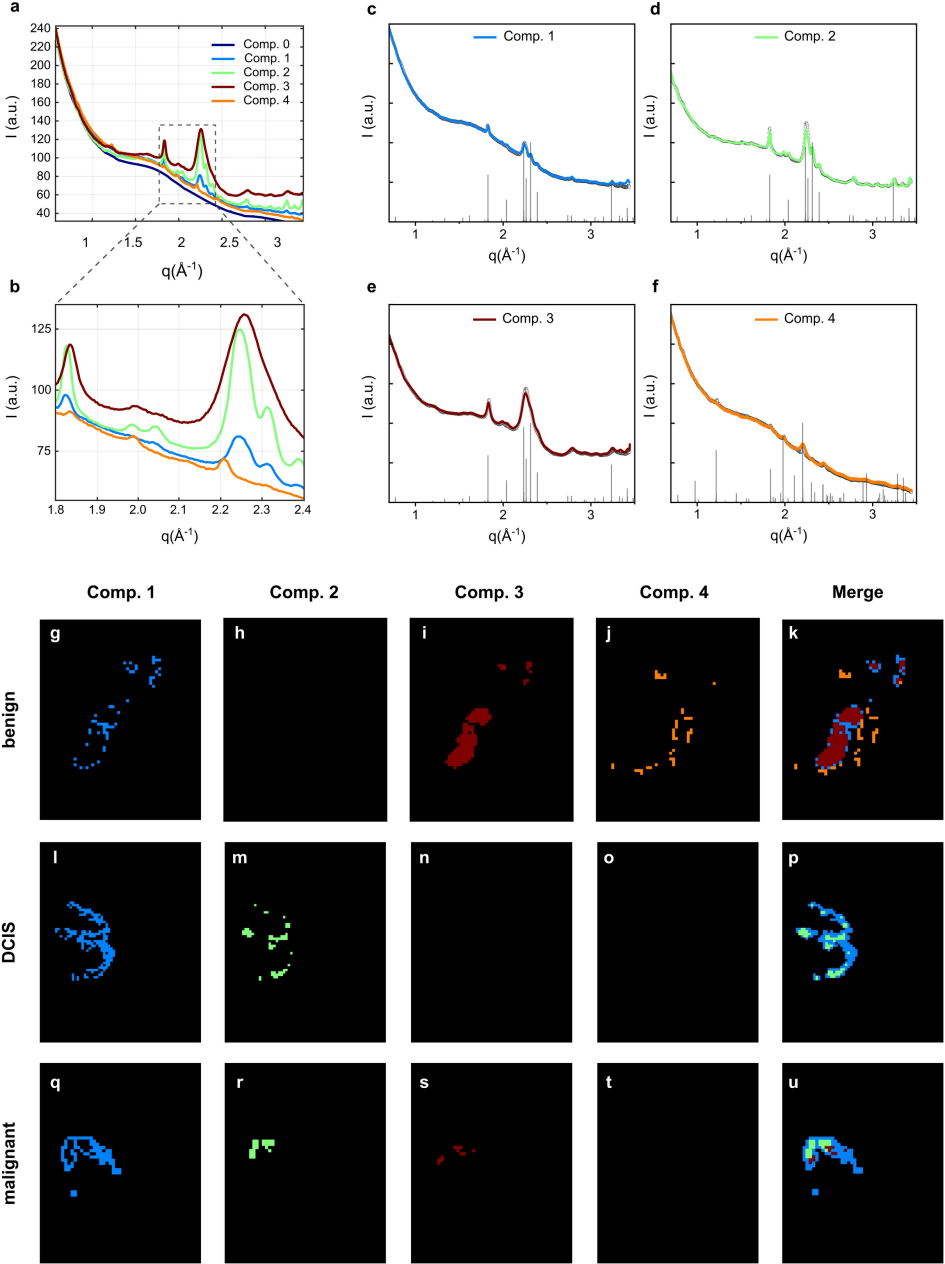
WAXS data collected on three breast tissue biopsies, all containing MC classified according to the level of malignancy (benign, DCIS, malignant/IDC). The 1D WAXS profiles, shown in (*a*) and in (*b*) as a zoom-in on the region Δ*q* = 1.8–2.4 Å, refer to the selected components extracted after segmentation. Component 0 is the background while components 1–4 refer to the crystalline structures forming the MC: hy­droxy­apatite (HAP, blue/component 1, green/component 2 and red/component 3) and whitlockite (WHIT, orange/component 4), as determined after Rietveld analysis of classified WAXS signals and shown in (*c*)–(*f*). The WAXS microscopy images (*g*)–(*u*) after signal-classification analysis show the location of HAP and WHIT in the samples [with colour coding identical to the spectra in (*c*)–(*f*)]. Explored area: 3 × 2 mm. Raw data in this figure have been partially discussed by Vanna *et al.* (2020 ▸) and Morasso *et al.* (2023 ▸).

Fig. 1 ▸ shows the WAXS data [1D profiles in Figs. 1 ▸(*a*)–1 ▸(*f*) and microscopy images in Figs. 1 ▸(*g*)–1 ▸(*u*)] collected on the three investigated breast tissues, all containing MC classified according to the level of malignancy (benign, DCIS, IDC).

The 1D WAXS profiles shown in Fig. 1 ▸(*a*), and in Fig. 1 ▸(*b*) as a zoom-in on the region Δ*q* = 1.8–2.4 Å, refer to the selected components extracted after a signal-classification analysis of the entire WAXS dataset was performed (Lutz-Bueno *et al.*, 2018 ▸). This analysis resulted in four predominant WAXS intensity profiles being identified, shown in Figs. 1 ▸(*c*)–1 ▸(*f*). Apart from component 0, which is the background, components 1–4 identify the crystalline structures forming the MC. The four WAXS profiles selected via the classification analysis were quantitatively analysed using the Rietveld approach, and the main refined parameters (unit-cell dimensions *a*, *b* and *c*, and crystalline domain size along specified crystallographic directions) are reported in Table 1 ▸. Three of the four profiles, marked in green, dark red and blue, were indexed as scattering profiles of the HAP crystal structure [hexagonal Ca_5_(PO_4_)_3_(OH), ICSD #187840] with different degrees of crystallinity and abundance: low-abundance and high-crystallinity HAP [blue profile in Fig. 1 ▸(*c*), component 1], high-abundance and high-crystallinity HAP [green profile in Fig. 1 ▸(*d*), component 2], and high-abundance and low-crystallinity HAP [dark red profile in Fig. 1 ▸(*e*), component 3]. With ‘crystallinity’ we refer to the domain size, as also listed in Table 1 ▸, *i.e.* the degree of long-range order on the atomic scale, with high crystallinity and thus large domain size leading to narrow diffraction peaks. With ‘abundance’ we refer to the strength of the WAXS signal within one pixel of the sample scan, correlated to the abundance of crystalline HAP within that sample volume. The fourth WAXS profile identified by the signal-classification analysis, shown in orange in Fig. 1 ▸(*f*) (component 4), was indexed as the scattering profile of crystalline WHIT (rhombohedral Ca_9.5_MgO_28_P_7_, COD #9012137).

Here, we summarize the results of the WAXS investigation (colours refer to Fig. 1 ▸):

(i) All samples exhibit MC with the HAP crystal structure.

(ii) The benign sample exhibits extended areas of low-crystallinity and high-abundance HAP (dark red), while the malignant sample does in much smaller areas.

(iii) The DCIS and IDC samples exhibit mainly high-abundance and high-crystallinity HAP (green) and low-abundance and high-crystallinity HAP (blue).

(iv) Only the benign sample exhibits WHIT (orange), which is mainly located in a rim around the low-crystallinity high-abundance HAP variant (dark red) area.

The SAXS microscopy data identify collagen with different lattice spacings (*D*): *D* = 61.6 nm and *D* = 63.0 nm. Sample-preparation steps like formalin treatment, paraffin embedding, ethanol immersion and drying affect (typically shorten) the collagen *D* spacing. We speculate that mineralized collagen is stabilized (like, for example, bone and teeth, where the imprint of the collagen *D* spacing in the bio-mineralization is observable after harsh treatments, and even in fossils) and thus less prone to pronounced changes of the *D* spacing (Gourrier *et al.*, 2017 ▸). This would explain why the collagen with the larger *D* spacing coincides with the HAP mineralized area: the mineralization may prevent partially the pronounced shrinkage occurring in soft tissue during the sample-preparation procedure employed here. Thus, while our findings are in agreement with earlier reported higher *D*-spacing values near malignant tissue (Conceição *et al.*, 2009 ▸), we refrain from any interpretation in the context of MC here due to the potential for being a mere artefact of the sample preparation.

### 4.2. XRF mapping and spectroscopy

**Figure 2:**
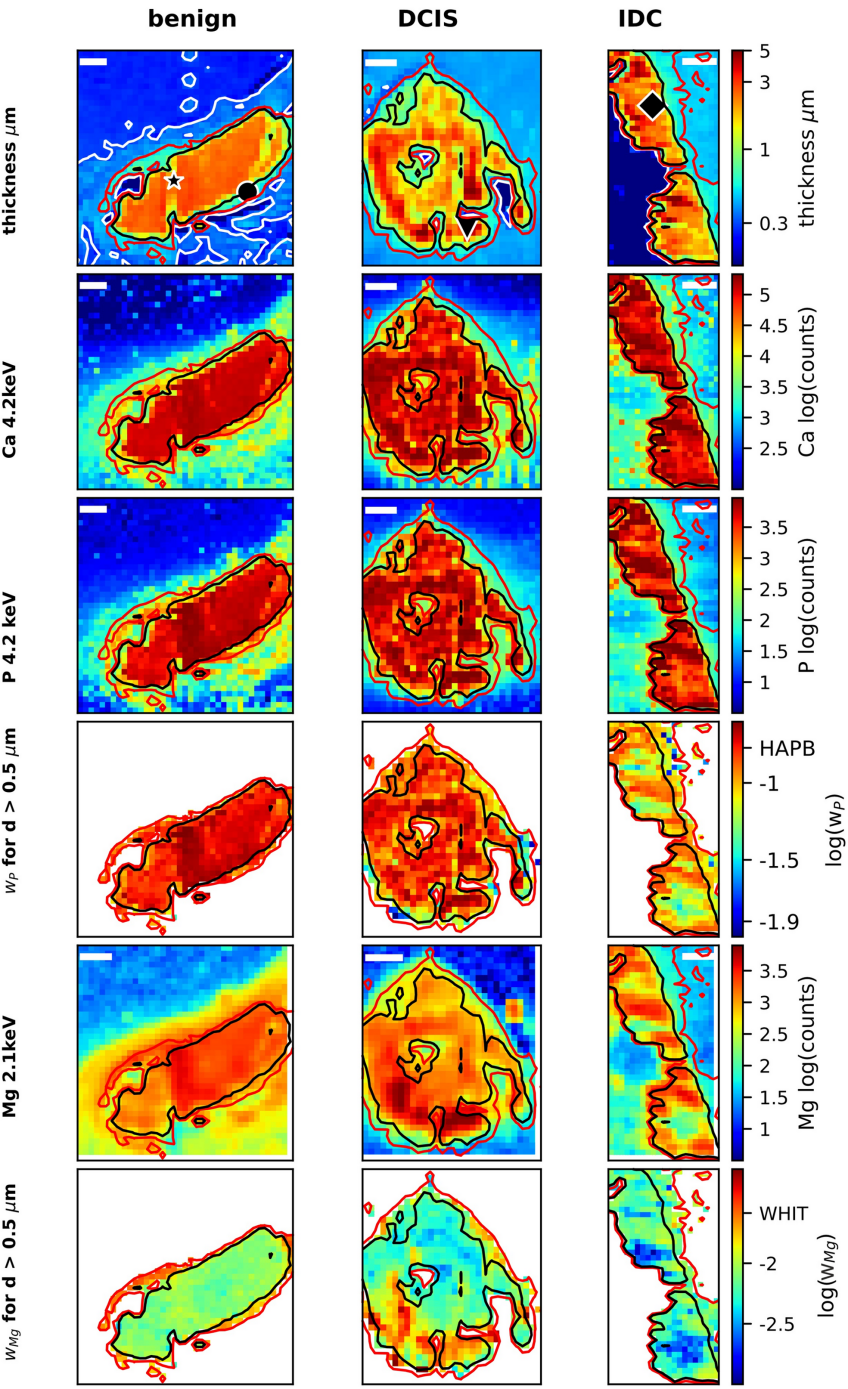
XRF analysis of MC: effective sample thickness and XRF analysis for the benign sample (first column), DCIS (middle column) and IDC (right column). Row 1: effective sample thickness as derived from X-ray absorption at 2.5 keV. Three contour lines mark the effective sample thicknesses of 0.3 µm (white), 0.5 µm (red) and 1 µm (black) calculated under the assumption of HAP type B material being imaged. The lines for 0.5 and 1 µm effective thickness are added to all other plots in this figure. A black star, circle, triangle or diamond shape marks the locations where the XRF spectra shown in Fig. 3 ▸ were recorded. Row 2: fluorescence intensity map for Ca taken at 4.2 keV. Row 3: fluorescence intensity map for P taken at 4.2 keV. Row 4: P mass-fraction map as calculated from XRF data taken at 4.2 keV. Row 5: fluorescence intensity map for Mg taken at 2.1 keV. Row 6: Mg mass-fraction map derived from data taken at 2.5 keV. The intensity maps for P (4.2 keV) and Mg (2.1 keV) relate to the same sample and measurement at 2.5 keV as presented in Fig. S2 of Morasso *et al.* (2023 ▸). The Mg mass fraction (Row 6) data are also shown in Fig. 1(*a*) of Morasso *et al.* (2023 ▸). Note the different scaling of data in the different panels. Scale bars: 100 µm.

Here, we present a detailed quantitative analysis of XRF spectra taken from MC, using XRF data presented by Morasso *et al.* (2023 ▸) combined with additional data taken at 2.1 and 4.2 keV. We show the complete data analysis in full detail to further justify the main conclusions and to discuss potential pitfalls in the data analysis of such systems. Furthermore, we show the presence of elements other than Mg in MC and make a quantitative analysis of the different elements. Using the effective sample thickness, raw XRF intensities and derived mass fractions for Ca, P and Mg (Fig. 2 ▸) we illustrate the spatial distribution of these elements in the MC, demonstrate the presence of Mg inside the MC and show its enhanced mass fraction in the outer rim of the MC. The XRF point spectra (Section 4.2.2) demonstrate the necessity of using different excitation energies to extract information on both the dominant elements in the heavy sample matrix (P, Ca) and the light trace elements (Mg, Na), and unambiguously show the presence of the light elements inside the MC. We then show the spatial distribution of biologically relevant elements (O, Na, S, Cl) inside the MC, in terms of both absolute intensity (Fig. 4) and mass fraction (Fig. 5). Finally, in Figs. 6 and 7, we investigate systematic trends between sample composition and thickness, to corroborate the observed enhancement of Mg in the outer rim of the MC for the benign sample.

#### 4.2.1. Effective sample thickness

The effective sample thickness (first row in Fig. 2 ▸) of the MC is 3–5 µm, as estimated from the X-ray absorption of photons at an energy of 2.5 keV. These measurements identify the location of the MC. While the major MC of the benign sample are quite flat, the other two samples exhibit significant effective thickness variations within their MC, and these areas are surrounded by a region of ∼0.5–1 µm effective thickness. The thickness estimate assumes a matrix of HAP type B. Hence, regions with a matrix of lower optical density (organic tissue, or the embedding paraffin, or a mixture of crystals with different X-ray absorptions) will have a larger physical thickness compared with the effective sample thickness derived from the optical density. For details of calculations, see Section 3.2.3 and Section S1.3.

#### 4.2.2. XRF spectra

**Figure 3:**
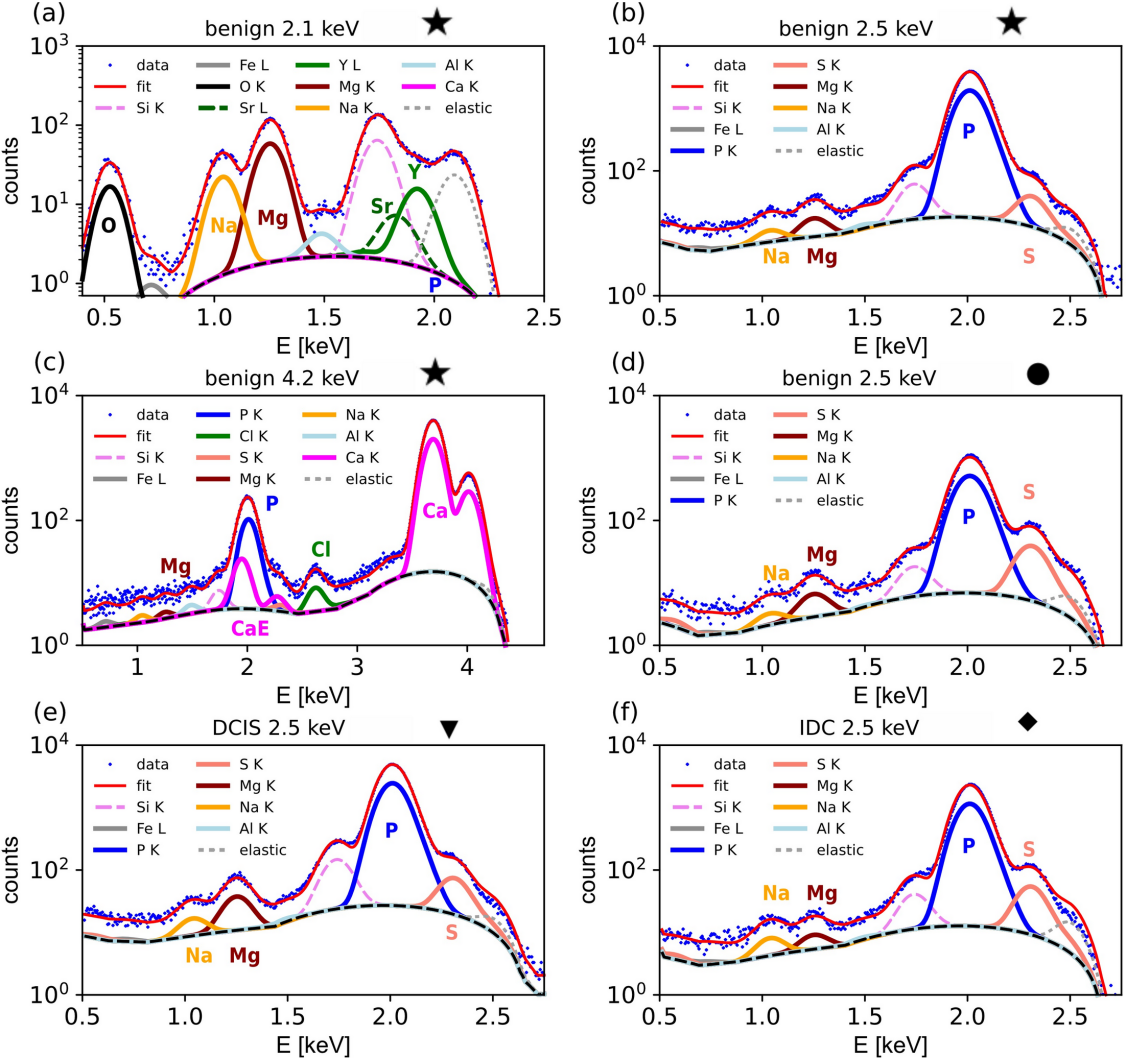
XRF spectra (blue dots) taken at different conditions and positions as marked in Fig. 2 ▸. (*a*)–(*c*) XRF spectra from the centre position of the benign sample, marked with a star in Fig. 2 ▸, recorded at 2.1, 2.5 and 4.2 keV, respectively. (*d*) XRF spectra from an edge position of the benign sample, marked with a circle in Fig. 2 ▸, recorded at 2.5 keV. (*e*) XRF spectra of the DCIS sample, with position marked as a triangle in Fig. 2 ▸, recorded at 2.5 keV. (*f*) XRF spectra of the IDC sample, with position marked as a diamond in Fig. 2 ▸, recorded at 2.5 keV. The red line shows the XRF intensity calculated from the fitted model. The modelled emission lines for the different elements, elastic scattering peaks and estimated background are shifted vertically (*i.e.* divided by a factor of 2) to ease visibility of these peaks in the figure. For colour code, see figure legend. Some important peaks are explicitly marked; CaE marks the location of the escape peak for Ca. Data taken at 2.1 keV average four adjacent pixels, while data at 2.5 and 4.2 keV are spectra from single pixels.

Fig. 3 ▸ compares typical XRF spectra taken from different samples and locations, as marked on the plotted effective thickness in Fig. 2 ▸, row 1. Figs. 3 ▸(*a*)–3 ▸(*c*) show XRF spectra from the same location in the centre of the benign sample (marked as a star in Fig. 2 ▸), recorded at excitation energies of 2.1, 2.5 and 4.2 keV, respectively. The XRF spectrum recorded at the lowest energy [Fig. 3 ▸(*a*), 2.1 keV] unambiguously shows the presence of O, Na and Mg (emission lines at 0.52, 1.04 and 1.25 keV, respectively) in the MC. The emission line at ∼1.7 keV mainly refers to Si, which is a contamination from the Mylar supporting foil (see Section S1.4). The region between the emission line of Si (1.74 keV) and the elastic scattering (2.1 keV) can only be fitted if the *L* lines of Sr (*L*α_1_: 1.806 keV) and Y (*L*α_1_: 1.923 keV) are considered in the fit (see Section S1.6). The spectra taken at 2.1 keV provide clear evidence for the presence of light elements. However, they are not sufficient to determine the mass fraction relative to the matrix elements P and Ca, as these elements are not excited.

The emission line of the matrix element P can be excited at 2.5 keV [Fig. 3 ▸(*b*), same location on the sample]. This spectrum now exhibits the dominant P *K*α emission line, which introduces a significant background at lower energies (see also Section 3.2.1). While this hampers the visibility of lighter elements, both Mg and Na remain detectable. Also, the emission line of S (2.3 keV) is visible, but it partially overlays with the elastic scattering peak at 2.5 keV and the P emission line. Hence, the spectra taken at 2.5 keV can be used to infer the Mg/P ratio, which is the base to derive the mass fraction of Mg in the microcalcification matrix.

To derive the P mass fraction in the MC, both P and Ca need to be excited. Fig. 3 ▸(*c*) shows an XRF spectrum taken at 4.2 keV from the same location. This spectrum serves well to determine both Ca and P content, but any signal from light elements (O, Na, Mg…) is masked by the noise level, making the analysis of light elements impossible. Only the stronger emission line of Cl at 2.6 keV remains visible. Both the P and S emission lines (2.3 keV) overlap with the two escape lines [marked as CaE in Fig. 3 ▸(*c*)], which are artefacts of the Si fluorescence detector, generated by the Ca *K*α and Ca *K*β lines, respectively. Hence, peak deconvolution using *PyMCA* is essential to analyse the amount or mass fraction of both S and P.

The XRF spectrum in Fig. 3 ▸(*d*) is taken at the edge of the MC in the benign sample (position marked as a filled circle in Fig. 2 ▸) and shows a lower intensity of the P emission line compared with a location inside the calcification [Fig. 3 ▸(*b*)]. Most importantly, both spectra clearly show the presence of Mg. Finally, Figs. 3 ▸(*e*) and 3 ▸(*f*) show XRF spectra taken at 2.5 keV from the DCIS sample and the IDC sample, respectively, showing the same features as the spectra taken from the benign sample.

These three spectra illustrate the challenge for the quantitative analysis. Data taken at 4.2 keV provide the Ca/P ratio, and hence the matrix composition, but are not useful to study the light elements. Spectra taken at 2.5 keV energy excite both P and some light elements, in particular Mg and Na, allowing one to infer the mass fractions of these elements. Finally, only the excitation at 2.1 keV, below the P *K* edge, enables recording of the very weak peaks of O, Fe, Sr and Y, which are invisible using higher excitation energies. Hence, employing different excitation energies significantly improves the XRF analysis.

#### 4.2.3. Intensity maps of Ca and P

Rows 2 and 3 in Fig. 2 ▸ show the intensity maps for Ca and P, both taken at an excitation energy of 4.2 keV. Regions of high fluorescence of Ca and P correlate with the region of maximum X-ray absorption. Also, regions with effective thickness estimates of more than 0.5–1 µm (row 1) coincide mostly with the XRF of both Ca and P, corroborating the presence of both Ca and P also in these regions of the sample, as expected for the presence of HAP or WHIT.

The fluorescence signal of Ca shows a weak ‘halo’ surrounding the sample, mostly in regions with an effective thickness below 0.5 µm. The intensity is of the order of ∼1% or less of the main signal of the MC and could be an artefact from scattered photons in the tails of the focused beam, which excites the highly concentrated sample. We will therefore refrain from interpreting regions of the maps where the effective thickness is below 0.5 µm.

#### 4.2.4. P mass-fraction maps

Both the Ca and P signals vary in the MC, as can be seen in the intensity plots in rows 2 and 3 of Fig. 2 ▸. The estimated P mass fraction (Fig. 2 ▸, row 3) in the MC ranges from 0.12 to 0.25, roughly consistent with the expected value of 0.169 for P in a HAP type B matrix or WHIT (∼0.18). As further discussed in Section S1.5.1, we estimate the absolute error in calculated mass fraction to be less than 20–30%. Hence, the estimated mass fractions are consistent with the presence of both HAP and WHIT, but we cannot use the mass fraction to distinguish between the two phases. The variations in P mass fraction (Fig. 2 ▸, row 4) seem to partially correlate with the effective thickness measurements for the benign and DCIS samples, but less for IDC (row 1 and row 4), which may reflect the impact of local geometry variations on the analysis. This partial correlation may reflect effects of local corrugations of the surface. Given these uncertainties, care must be taken when using only the maps shown in Fig. 2 ▸ to draw a firm conclusion as to whether the P mass fraction is indeed slightly non-homogeneous inside the MC. However, two further lines of evidence support an inhomogeneity of the P mass fraction (or the P/Ca atomic ratio). First, the XANES spectra (Section 4.3) would be consistent with the partial presence of amorphous calcium species (AC) such as amorphous calcium carbonate (ACC) in the sample itself. Secondly, the P mass fraction seems to quite systematically vary with the optical density and hence the effective thickness (see Section 4.2.8). This would be consistent with local variations of the P mass fraction inside the MC, as shown in row 4 in Fig. 2 ▸.

#### 4.2.5. Mg spatial distribution and mass fraction

The Mg intensity maps for the three samples (Fig. 2 ▸, row 5) were derived from data taken at 2.1 keV, because at this excitation energy we can unambiguously identify the Mg fluorescence peak [see Section 4.2.2 and Fig. 3 ▸(*a*)]. The maps clearly show the presence of Mg both in the MC and in regions of effective thickness between 0.5 and 1 µm, *i.e.* outside but next to the main microcalcification areas. Mg maps derived from data at 2.5 and 2.1 keV look practically identical, and hence the 2.5 keV (not shown) data are used to derive the mass fraction of Mg (Fig. 2 ▸, row 6).

For the benign and IDC samples, the Mg mass fraction inside the MC is of the order of 0.01, while the DCIS sample shows strong variations with peak values of 0.03. For the benign sample, there is evidence also of an enhanced mass fraction in the outer rim. Both Ca and P are detected in the rim (Fig. 2 ▸, rows 2 and 3), and the P mass fraction (Fig. 2 ▸, row 4) in this region is consistent with the existence of HAP and WHIT. Similarly, the Mg mass fractions in the rim of about a few percent are consistent with the Mg mass fraction in WHIT.

We are aware that quantitative analysis of XRF data can suffer from experimental pitfalls but, on the basis of the considerations presented in Section S1.5, we conclude that the enhancement of Mg in the rim region is indeed a robust result.

#### 4.2.6. XRF intensity plots of other elements

**Figure 4:**
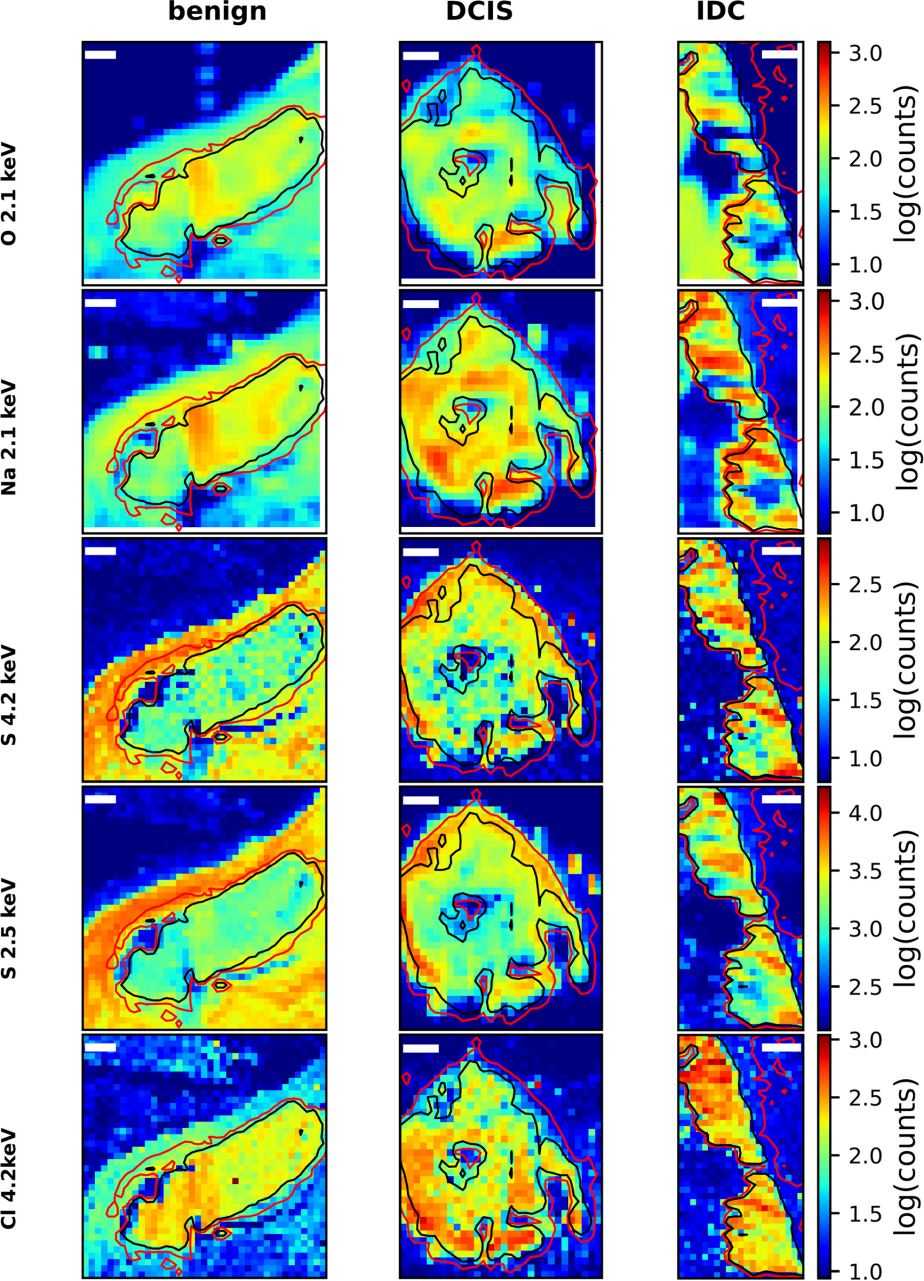
Intensity plots of other biologically relevant elements in the three samples. Row 1: O measured at 2.1 keV. Row 2: Na measured at 2.1 keV. Row 3: S measured at 4.2 keV. Row 4: S measured at 2.5 keV. Row 5: Cl measured at 4.2 keV. Isolines refer to effective sample thicknesses of 0.5 µm (red) and 1 µm (black), see also Fig. 2 ▸. Scale bars: 100 µm.

The XRF spectra displayed in Fig. 3 ▸ show that, besides Ca, P and Mg, other trace elements are found in the MC, most notably O, Na, Cl and S. The intensity map in Fig. 4 ▸, row 1, shows the spatial distribution of oxygen, which is part of the HAP matrix. We refrain from further interpretation in the region surrounding the MC because the O emission line is only partially recorded, and it is present in the support foil. Fig. 4 ▸, row 2, shows the distribution of Na, which is also clearly present in all samples. Rows 3 and 4 of Fig. 4 ▸ show the distribution of S as derived from data taken at 4.2 and 2.5 keV, respectively. Potential peak deconvolution artefacts should be fundamentally different for maps taken at 2.5 keV (interference with elastic scattering, and dominant P line) and at 4.2 keV (interference with the escape peak of Ca). As maps taken at these two energies show a more or less identical distribution of S in the sample, this result is trustworthy and confirms the presence of S in the MC. Similarly, we can confirm the presence of Cl in the MC (Fig. 4 ▸, row 5). These results clearly show that the MC are not pure crystals but contain small amounts of biologically relevant ions.

#### 4.2.7. Mass-fraction maps of other trace elements

**Figure 5:**
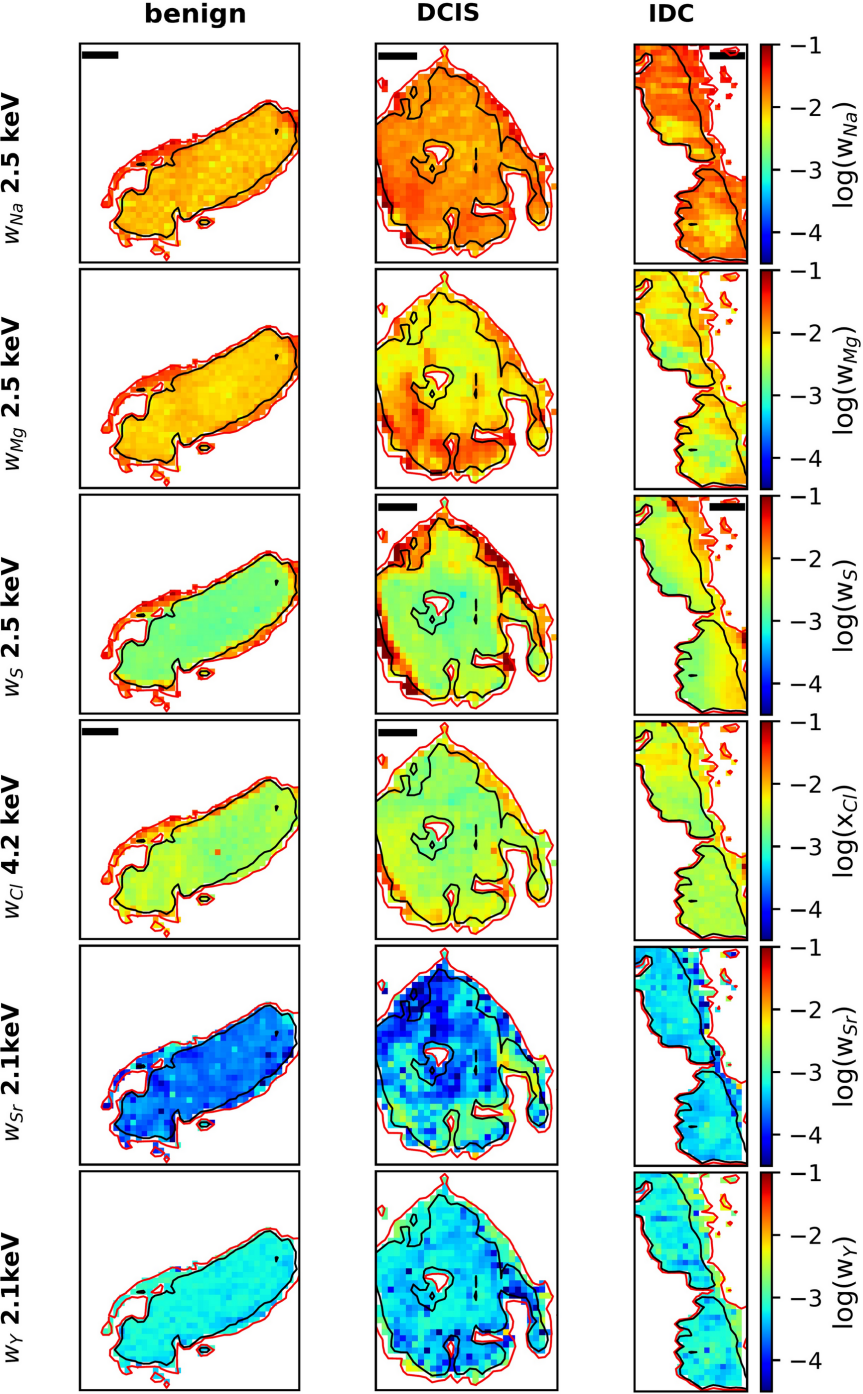
Mass fractions for various trace elements found in the three samples. For Na, Mg, Cl and S, XRF spectra were fitted for each individual pixel. For Sr and Y, each pixel is the the average of four adjacent pixels. Isolines refer to effective sample thicknesses of 0.5 µm (red) and 1 µm (black), see also Fig. 2 ▸. Scale bars: 100 µm. The mixing ratio for Mg is replotted from Fig. 2 ▸, to allow direct comparison on the same scale between all data here.

Fig. 5 ▸ shows the mass fractions of Na, Mg, S, Cl, Sr and Y as maps. For details of calculations, see Section S1.6. The maps demonstrate that all trace elements can be found in the MC, with Na and Mg at roughly equal levels in the benign sample, but with an enhanced Na mass fraction in the DCIS and IDC samples relative to the Mg mass fraction. S is also found inside the MC. Its mixing ratio in the HAP type B matrix seems to be enhanced in the region surrounding (*i.e.* in regions with effective thickness between 1 and 0.5 µm) the MC for the benign and DCIS samples, but less so for the IDC sample. The distributions of the Na, Mg, S and Cl mixing ratios in Fig. 5 ▸ (rows 1–4) are different for the different elements, which excludes that the observed elemental distributions are artefacts due to inhomogeneities of the sample surface geometry. For further discussion of potential artefacts, see Section S1.5.1. Finally, the mixing ratios of Sr and Y are the lowest of the elements studied here. On average, there is more Y than Sr.

#### 4.2.8. Mass-fraction analysis by size binning

**Figure 6:**
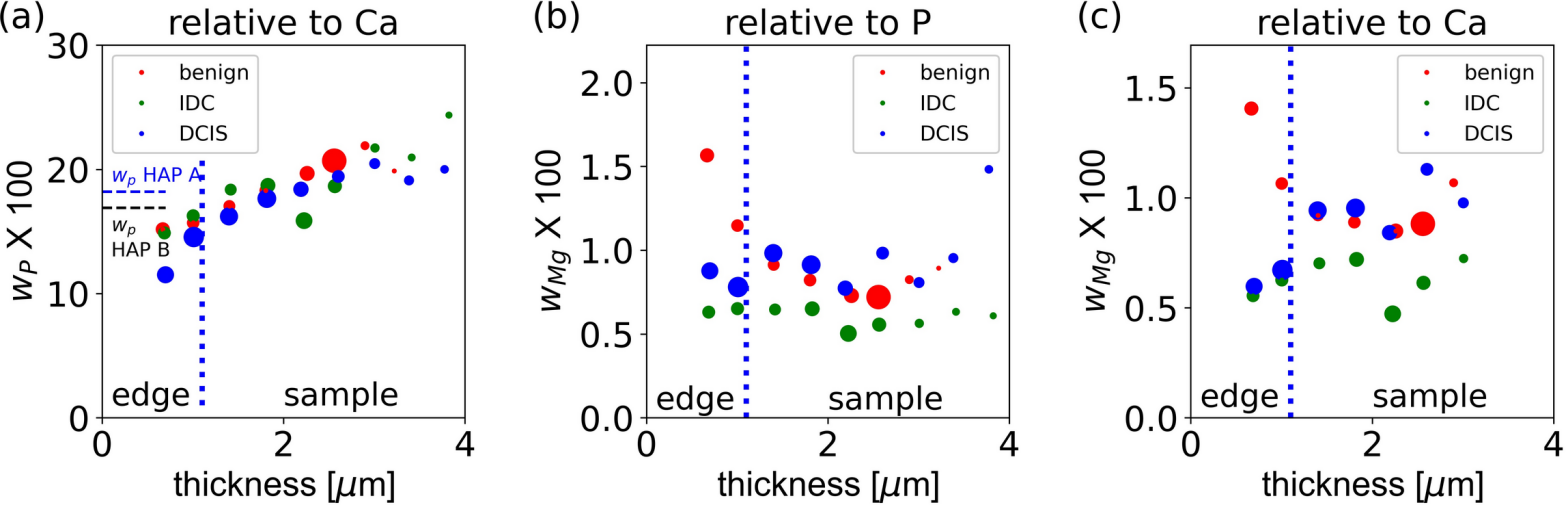
Estimated mass fraction as a function of effective sample thickness, calculated for an exit angle of 6° of the sample surface relative to the detector. The vertical dotted solid line roughly marks where the edge (∼1.1 µm) of the microcalcification region of the sample is located. (*a*) P mass fraction as derived from data taken at 4.2 keV, assuming a theoretical mass fraction for Ca of 0.39. For comparison, the theoretical mass fractions for HAP type A and type B are marked as horizontal blue and black lines, respectively. (*b*) Mg mass fraction derived from data at 2.5 keV, assuming that P has the theoretical mass fraction of 0.196. (*c*) Mg mass fraction derived from data at 2.5 and 4.2 keV, assuming that Ca has the theoretical mass fraction of 0.39. For further technical details, see Section S1.5.2.

Fig. 6 ▸ shows the estimated P and Mg mass fractions for all three samples as a function of the effective sample thickness, as derived for a scattering angle of 6°, using the methodology described in Section 3.2.5. The P mass fraction [Fig. 6 ▸(*a*)] is derived from data taken at 4.2 keV, assuming a theoretical mass fraction for Ca of *w*_Ca_ = 0.39. The determined P mass fraction [Fig. 6 ▸(*a*)] seems to follow a linear trend as a function of effective thickness for all samples. The XANES spectra discussed in Section 4.3 indicate that the samples are not pure calcium phosphate but may also contain calcium carbonate, consistent with the data published by Vanna *et al.* (2020 ▸). Because the X-ray absorption of calcium carbonate is lower than that of calcium phosphate, an enhanced calcium carbonate fraction will increase the X-ray absorption, causing a lower estimate for the effective thickness. Hence, the observed trend would be consistent with the presence of calcium-containing P-free species (*e.g.* carbonate) in the sample. Fundamentally, the effective P/Ca mixing ratio *x*_P,eff_ in a mixture of HAP (mixing ratio *x*_P,HAP_) with fraction α of a P-free but Ca-containing species (*e.g.* CaCO_3_) would depend linearly on the fraction α: *x*_P,eff_ = (1 − α)*x*_P,HAP_. Because the optical density depends linearly on the sample composition (*i.e. α*), the effective mixing ratio of P (and hence, in good approximation, the P mass fraction) would also depend linearly on effective thickness, in qualitative consistency with the trend observed in Fig. 6 ▸(*a*). However, because quantitative modelling is hampered by the potential presence of additional species, most notably WHIT, we refrain from quantitative modelling.

For comparison, the theoretical mass fractions of P in HAP type A (18.2%, which roughly equals that of WHIT, 18.4%) and HAP type B (16.9%) are indicated as horizontal dashed lines. These numbers are consistent with an estimated P mass fraction within 10–20%. As the absolute value is very sensitive to systematic errors in the sample geometry (see Section S1.5), there is good consistency of the experimentally determined P mass fraction with the presence of HAP species.

The Mg mass fraction is derived from the XRF data taken at 2.5 keV, assuming a theoretical mass fraction for P of *w*_P_ = 0.196 [Fig. 6 ▸(*b*)] and combining XRF data taken at 4.2 and 2.5 keV using a theoretical mass fraction for Ca of *w*_Ca_ = 0.39 [Fig. 6 ▸(*c*)]. The calculations for the Mg mass fraction in both Figs. 6 ▸(*b*) and 6 ▸(*c*) show the following general trends. First, the Mg mass fraction is enhanced in regions of lower effective thickness for the benign sample, but not for the IDC and DCIS samples. Secondly, the Mg mass fraction in the IDC sample is the lowest. Both trends are visible in both Figs. 6 ▸(*b*) and 6 ▸(*c*), independent of using P or Ca as the reference element, and hence are not artefacts induced by the trend of the P mass fraction [Fig. 6 ▸(*a*)]. Furthermore, the expected mass fraction in WHIT should be 2.3%, ∼30% higher than the estimated mass fraction of Mg [Fig. 6 ▸(*b*)] in the outer part of the benign sample. This is in qualitative consistency with the model that there is some WHIT in the outer part of the benign sample.

**Figure 7:**
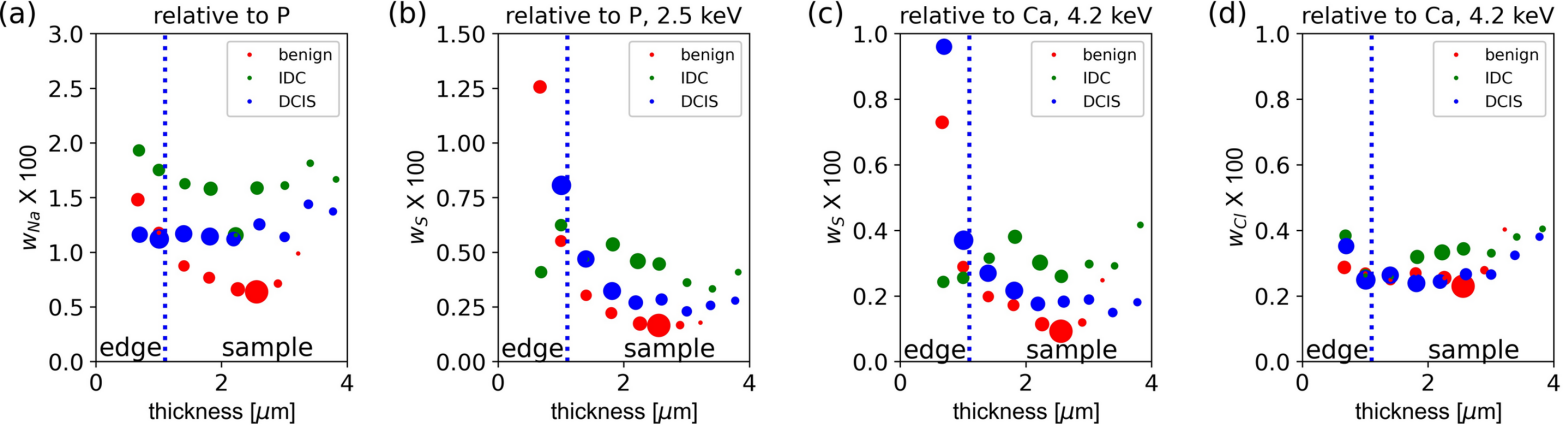
Estimated mass fraction as a function of effective sample thickness. (*a*) Na mass fraction derived from data taken at 2.5 keV, assuming that P has the theoretical mass fraction of 0.196. (*b*) S mass fraction derived from data at 2.5 keV, assuming that P has the theoretical mass fraction of 0.196. (*c*) S mass fraction derived from data at 4.2 keV, assuming that Ca has the theoretical mass fraction of 0.39. (*d*) Cl mass fraction derived from data at 4.2 keV, assuming that Ca has the theoretical mass fraction of 0.39.

Fig. 7 ▸ shows the estimated mixing ratios for Na, S and Cl, calculated for different HAP thicknesses and size bins. The mass fraction of Na is ∼0.005–0.02, with the malignant sample showing the highest mass fraction of Na, except for the data taken at the lowest effective thickness. When the S mass fractions are plotted as a function of effective thickness for the data taken at 2.5 and 4.2 keV [Figs. 7 ▸(*b*) and 7 ▸(*c*)], both datasets show the same trends: the malignant sample has the lowest mass fraction for S compared with the others, except for regions of lowest effective thickness (*i.e.* in the periphery of the sample), with all samples exhibiting the highest S mass fractions in their thinner parts. This is fully consistent with the mass-fraction map for S (Fig. 5 ▸, row 3), which shows an enhanced S rim in the thinner parts surrounding the MC. The S mass fraction as determined relative to P (2.5 keV excitation energy) and relative to Ca (4.2 keV excitation energy) is for both methods comparable, of the order of 0.001–0.01. Finally, we find Cl with a mass fraction of ∼0.002–0.004 in all samples.

### 4.3. XANES spectra

**Figure 8:**
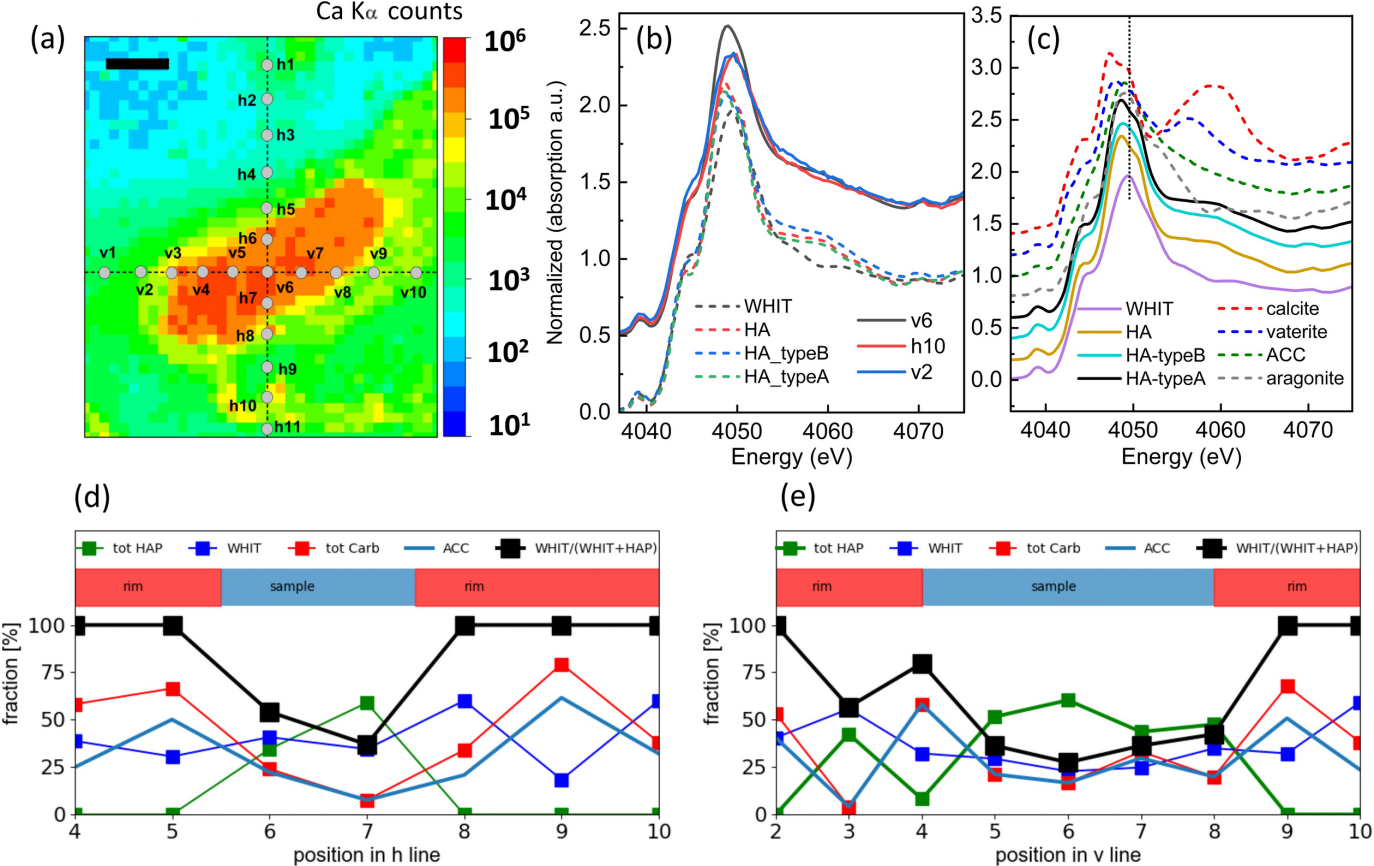
(*a*) XRF raster scanning of the benign sample using incident X-rays of 4.2 keV. The spatial distribution of the Ca *K*α fluorescence peak intensity is plotted on a logarithmic scale, and the positions where the XANES spectra have been acquired are marked on the map. The scale bar corresponds to 100 µm. (*b*) Absorption coefficient at the Ca *K* edge plotted for three points within the sample, as marked in the XRF plot. (*c*) WHIT, HAP and calcium carbonate including amorphous (ACC) reference spectra. The carbonate spectra are taken from Xto *et al.* (2019 ▸). (*d*), (*e*) Fractions of different species (expressed in percentage) as a function of locations for the lines marked as (*e*) h and (*d*) v derived from LCF for all apatite species (tot HAP = HAP + HAP A + HAP B), WHIT, calcite, AC, and the fraction of WHIT in the WHIT and apatite group [WHIT/(WHIT + tot HAP)].

X-ray absorption spectra at the Ca *K* edge were taken at different locations on the different samples using a microbeam of ∼30 × 30 µm in size. For these measurements, the samples were mounted at 45° relative to the incoming beam to maximize the overall fluorescence count rates. For the benign sample, a series of spectra were taken along a horizontal line (positions v1 to v10) and a vertical line (positions h1 to h10) crossing the centre of the MC (marked as dashed lines in Fig. 8 ▸). For the other two samples, XANES spectra have been recorded on specific thin and thick regions (see Section S2, and Figs. S11 and S12).

To demonstrate the main findings, we compare spectra taken at three different positions marked in Fig. 8 ▸(*a*), *i.e.* in position v6 at the centre of the MC and in v2 and h10 in the outer part of the MC. The reference spectra for HAP, HAP type A and HAP type B (dashed lines) are shown in Fig. 8 ▸(*b*) in comparison with the measured XANES spectra from the three locations in the sample (solid lines). In principle, the MC could also contain other Ca-containing biominerals, most notably calcium carbonates. Reference spectra for different calcium carbonates are shown in Fig. 8 ▸(*c*). To reduce artefacts from over-absorption, the reference powders were measured in TEY mode, whereas the spectrum from the centre (v6) was measured in transmission. The spectra taken in the outer part (v2 and h10) in partial fluorescence yield mode are effectively from optically very thin layers and hence are over-absorption free. Except for ACC, these spectra have key features that are found only in a few spectra taken outside the centre of the benign sample (Fig. S9, position h1), making the presence of crystalline carbonates unlikely in the MC of these samples. The reference spectra differ in two distinctive features: the white line for HAP is at ∼4048.6 eV while that for WHIT is shifted to a higher energy at ∼4049.5 eV. Furthermore, in the region around 4060 eV, all HAP reference spectra have an enhanced absorption coefficient in comparison with WHIT. There are no further distinct defining features for the different HAP phases, except for minor differences in white line intensity and shoulder height when comparing the different HAP polymorphs, which makes it difficult to identify the different HAP phases. Comparing the white line position of the reference spectra with those from the samples provides evidence for the presence of WHIT in the periphery of the MC (positions v2 and h10), but less in their centre (position v6).

For the benign sample, a series of spectra along two lines [marked as h and v in Fig. 8 ▸(*a*)] were taken. Figs. 8 ▸(*d*) and 8 ▸(*e*) show the fractions of the different species groups as derived from linear combination fitting (LCF) for positions where the effective thickness is larger than 0.5 µm. The blue and red upper parts mark positions taken in the sample and in the rim, respectively, as indicated in Fig. 8 ▸(*a*). We group the different species by plotting ‘tot HAP’ (= the sum of HAP, HAP A and HAP B fractions), WHIT, ACC and calcite. The quantity WHIT/(WHIT + tot HAP) reflects the fraction of WHIT in all Ca- and P-containing minerals. We did not include vaterite and aragonite in the fit, as these spectra have strong features [Figs. 8 ▸(*b*) and 8 ▸(*c*)], which are obviously not consistent with the XANES spectra from the MC. Also, AC carbonate or phosphate could be present in the sample. By the very nature of amorphous materials, their local structure is not well defined and is affected by the presence of water or organic matter. We do not have reference spectra of AC phosphate. Published spectra of AC phosphate (Zhang *et al.*, 2015 ▸) look similar to that of ACC that we use here but have a more pronounced pre-edge step. While the XANES spectra differ for amorphous materials, their main shapes are very similar. Therefore, we consider the ACC XANES spectrum as a reference to test for the presence of Ca ions in an amorphous environment (AC).

From Fig. 8 ▸, we can draw the following qualitative conclusions. First, HAP phases are found in the microcalcification region of the sample itself and less so in the rim region. Secondly, WHIT is found outside of the MC and, to a lesser extent, inside the MC. Third, as a consequence, WHIT dominates the fraction of calcium phosphates in the rim region. Finally, the spectra are consistent with a 10–25% fraction of carbonates in the microcalcification region of the sample and up to 50% in the rim with ACC being the dominant carbonate polymorph. The Ca XANES spectra from the DCIS and IDC samples taken at different points of effective thickness inside the MC region of the sample are shown in Figs. S11 and S12, respectively. There are no energy shifts observed nor any significant changes in the shape of the XANES spectra when comparing thick and thin regions in either of the MC. The measured Ca spectra (taken in fluorescence mode) resemble the HAP references rather than that of WHIT, as corroborated by the LCF summarized in Table S8 of the supporting information.

Furthermore, P *K*-edge XANES spectra have been acquired for the MC of the three samples, as well as on the HAP and WHIT powder standards (data not shown). All spectra indicate a tetrahedrally coordinated P atom, as expected in HAP. Upon comparison of spectra from different positions, it was observed that there were no differences in the characteristic spectral features and spectral shifts, except for a very slight variation in the white line intensity. While this may indicate slight changes in the electronic structure or in the symmetry of the tetrahedrally coordinated P atom, it could also be an artefact due to over-absorption (Carboni *et al.*, 2005 ▸; Noma, 1993 ▸) in these P-concentrated regions of the samples. We therefore refrain from further analysis of the P *K*-edge XANES spectra.

Although these conclusions are based on a limited dataset, there is significant internal consistency. First, the XRF model estimates the P mass fraction in the MC to be ∼12–20%, which is close to the theoretical value of 16.9% (18.2%) for the HAP (HAP type B) matrix. The weak, almost linear, dependence of the P mass fraction (or equivalently the P/Ca ratio) on the effective thickness, as reported in Fig. 6 ▸(*a*), is consistent with the presence of an additional species with a lower P/Ca ratio, in agreement with the presence of ACC in the MC suggested by the linear combination fits. The LCFs of the XANES spectra demonstrate the presence of some WHIT in the MC, consistent with the presence of Mg fluorescence in the MC. The dominance of WHIT in the rim region of the benign sample is consistent with our finding that the Mg mass fraction is enhanced in this region. Finally, enhancement of the WHIT phase around the MC in the benign sample was also found using Raman microscopy (Morasso *et al.*, 2023 ▸) and via the X-ray diffraction signal confirmed using WAXS.

## 5. Conclusions and outlook

XRF and X-ray absorption spectroscopy indicate Mg enrichment in a rim region surrounding the MC in the benign sample. By comparison with XANES reference spectra, this Mg-enriched calcified component has been identified as WHIT. The results of the WAXS analysis have confirmed the presence of WHIT in this rim region and have allowed the crystal structure parameters of this phase to be determined. Mg has been detected within the MC of all the samples, including the DCIS and IDC samples. However, the WAXS data indicate that it does not occur as a crystalline phase in these samples. The identification of ACC as a pr­oxy for amorphous Ca-containing phases as a significant component in the linear combination fits of the Ca *K*-edge XANES spectra for the MC in the benign sample substantiates the presence of amorphous phases in the MC. It is only possible to speculate that the differences in intra- and extracellular pH between healthy and cancer cells (Pérez-Herrero & Fernández-Medarde, 2021 ▸; White *et al.*, 2017 ▸) may be one of the origins of this phenomenon. In cancer cells, the glucose metabolism is altered, resulting in the production of more lactic acid. To prevent apoptosis, the protons are removed from the cell by upregulated proton pumps. As a result, the intracellular pH is higher (basic) than in healthy cells, whereas the extracellular pH is lower (acidic) than in the healthy state. This may result in a suppression of the formation of WHIT or even dissolution of previously formed WHIT in malignant neoplasms, as pH is a relevant parameter for the formation of WHIT (Jang *et al.*, 2015 ▸). Furthermore, it is possible that temporal or timing effects may play a role in this phenomenon. For instance, it has been reported that microcalcification formation increases with malignancy (Lev-Toaff *et al.*, 1994 ▸; Vidavsky *et al.*, 2018 ▸). This would mean as a possible consequence that, on average, some malignant MC are relatively newer, due to the presence of the tumour stimulating their new formation. Considering that the crystallization process is significantly slower for WHIT than for HAP and benign MC are significantly older than malignant MC, kinetic effects may be a contributing factor to WHIT occurring mainly in benign MC. The slow formation of WHIT may be attributed to a crystallization pathway involving intermediates such as brushite (Tas, 2016 ▸) or a low Mg ion concentration, as well as pH and temperature under physiological conditions.

The presence of calcium in an amorphous phase (AC) has been identified in the MC of the benign sample, with an increased concentration in a rim region of the MC. Consequently, both WHIT and AC are observed to occur predominantly (WHIT) or at least more frequently (AC) in this peripheral region of the MC. Although our data do not allow us to quantify the relationship between AC content and malignancy, they are nevertheless consistent with the previously reported decrease in content of carbonated calcium HAP with increasing malignancy (Baker *et al.*, 2010 ▸; Vanna *et al.*, 2020 ▸) and especially the occurrence of ACC mainly in benign MC and hardly in malignant MC (Vanna *et al.*, 2020 ▸).

The determination of the HAP structural parameters indicates the presence of a high-crystallinity and high-abundance HAP phase exclusively in the DCIS and IDC samples. This elevated crystallinity with increasing malignancy has been previously reported in other studies of MC in breast tissue (Gosling *et al.*, 2019 ▸) as well as our studies (Morasso *et al.*, 2023 ▸; Vanna *et al.*, 2020 ▸). Similar to what is reported above, about the effect of time in the difference between MC, it is possible to speculate as to whether the high crystallinity (and low carbonate substitution) is indicative of a faster formation process, as this could be in agreement with the observation of formation of new MC in the presence of malignancy (Lev-Toaff *et al.*, 1994 ▸; Vidavsky *et al.*, 2018 ▸). It is also unclear if differences in pH, or potentially in the degree of substitution or crystallization rate due to differences in the local chemical environment, are responsible for this difference in crystalline order.

The XRF data indicate that, in addition to Mg, the elements Na, S, Cl, Sr and Y are constituents of MC. The benign and DCIS samples appear to exhibit a region of enhanced S and Cl mass fraction surrounding the MC. Conversely, MC in IDC tissue demonstrated elevated S content at the core. It is anticipated that essential elements such as Na, S and Cl will be present in the human body. Strontium is also expected to be present in MC, as Sr can substitute for Ca in inorganic crystals and is a well known constituent of bones (Burton *et al.*, 1999 ▸). The element Y (yttrium), which is present in low quantities in biological systems, has not been fully elucidated in terms of its role or potential toxicity within these systems (He, 2013 ▸). Yttrium has been used to identify apatite in fossils that were embedded in carbonate minerals, and there is some evidence that yttrium appears to have a higher affinity for accumulation in apatite compared with carbonates (Gueriau *et al.*, 2018 ▸). This is consistent with our observation that there is more Y than Sr in the MC. Our XRF data on MC complement reports focusing on the soft tissue (Silva *et al.*, 2013 ▸; Conceição *et al.*, 2024 ▸).

Although the number of samples per condition is not statistically significant, the findings indicate a clear trend. For instance, they align with the WHIT assignment in confocal Raman scattering maps (Vanna *et al.*, 2020 ▸; Morasso *et al.*, 2023 ▸).

The data analysis employs a distinctive methodology, whereby the sample thickness, derived from the measured optical thickness, is utilized to further constrain the model for the XRF fitting. This process is conducted for each data point of the images, thereby accounting for local variations in the sample thickness or, alternatively, for variations in the matrix composition.

Our studies, performed on tissue from patients collected once the lesion is already formed in the tissue, do not allow us to demonstrate whether microcalcification features are only influenced by their health or tumoral environment or whether microcalcification features may also influence tumorigenesis. To demonstrate a role of microcalcification composition and structure in the formation and evolution of tumours, microcalcification features should be prospectively monitored in patients to evaluate how their composition and structure correlate to the formation and evolution of new malignant lesions. Gosling *et al.* (2023 ▸) recently reported microcalcification crystalline features as potential biomarkers of tumour recurrence but without demonstrating a role of MC in the formation or evolution of cancer. At the same time, a few *in vitro* studies reported that the amount and features of HAP (*e.g.* carbonate substitution) may amplify pathological processes in cultured breast cancer cells (Choi *et al.*, 2015 ▸; Morgan *et al.*, 2001 ▸). A more recent *in vitro* study reported that different synthetic minerals resembling breast MC have a certain impact in the evolution of *in vitro* growth of breast cancer multicellular spheroids (Cohen *et al.*, 2023 ▸). The authors tested the influence of two different forms of apatite [*i.e.* low-carbonate apatite (LCA) and high-carbonate apatite (HCA)] and a form of calcium oxalate. The role of Mg and WHIT, and the role of crystallinity, were not investigated. Their results suggest that HCA and LCA triggered cancerous behaviour in their models and that LCA induced a more pronounced promotion of precancer malignancy. As mentioned above, the presence of higher levels of low-carbonated apatite in malignant lesions has been observed in tissue samples from patients (Vanna *et al.*, 2020 ▸; Baker *et al.*, 2010 ▸) and this is coherent with the results of *in vitro* studies reported by Cohen *et al.* (2023 ▸). Also, as previously stated in the *Introduction*[Sec sec1], the presence of mineral deposits usually named ‘psammoma bodies’ is also characteristic of other types of cancers such as ovarian and thyroid cancer (Porcelli *et al.*, 2023 ▸). The study of psammoma bodies is however much less developed and we do not know if our findings replicate in other tumours.

Further studies, possibly using *in vivo* models and/or prospective studies on patients, will likely help clarify whether microcalcification features are a consequence, a cause, or somewhere in between when it comes to lesion malignancy.

We hope that these findings contribute to an understanding of the formation mechanisms of MC and thereby to the development of future diagnostic tools.

## 6. Related literature

The following reference is only cited in the supporting information for this article: Henke *et al.* (1993 ▸).

## Supplementary Material

Supporting information. DOI: 10.1107/S1600576724011750/ei5124sup1.pdf

## Figures and Tables

**Table:** The listed colours of the components refer to the WAXS signals shown in Fig. 1 ▸.HAP.

Component	*a* = *b* (Å)	*c* (Å)	Domain (Å) along [002]	Domain (Å) along [110]
Blue	9.3897	6.8652	244	129
Green	9.3897	6.8652	244	129
Dark red	9.3897	6.8652	96	53

**Table:** WHIT.

Component	*a* = *b* (Å)	*c* (Å)	Domain (Å)
Orange	10.3211	36.9674	46
