# Supplementary material for: Microcalcifications in breast cancer tissue studied by X-ray absorption, emission, scattering and diffraction
Source: J Appl Crystallogr. 2025 Feb 1;58(Pt 1):233–50. doi: 10.1107/S1600576724011750 (PMC11798518; doi:10.1107/S1600576724011750)
Supplement: Supplementary file 1 [file j-58-00233-sup1.pdf]

JOURNAL OF
APPLIED
CRYSTALLOGRAPHY

Volume 58 (2025)

Supporting information for article:

Microcalcifications in breast cancer tissue studied by X-ray absorption, emission, scattering and diffraction

Thomas Huthwelker, Camelia N. Borca, Davide Altamura, Liberato De Caro, Renzo Vanna, Fabio Corsi, Carlo Morasso, Greta Banfi, Giovanni Arpa, Oliver Bunk and Cinzia Giannini

S1. Data analysis procedures for XRF

S1.1. Absorption length and fluorescence energies of X-rays

A challenge for quantitative analysis of X-ray fluorescence arises from the differing X-ray absorption lengths for the various energies involved. In a crystal of HAP, the three selected excitation energies exhibit a remarkably similar probing depth δ of around 5 µm (for 2.1 keV: δ=5.3 µm, for 2.5 keV δ=5 µm, for 4.2 keV δ=5.1 µm). This value is approximately equal to the sample thickness, which results in a reduction in the intensity of the incoming photons by approximately a factor of 1/e along the photon's trajectory through the sample. In contrast, the absorption length of the emitted fluorescent photons in the crystal ranges from 1.5-18 µm (See Table S1). The maps were measured close to grazing exit conditions (exit angle ~6°). This geometry is only somewhat susceptible to surface effects and therefore minimizes the effect of errors induced by variations in sample thickness.

Table S1 Excitation and fluorescence energies of elements of interest compared with the X-ray absorption length in HAB type B. The absorption length is calculated as implemented in the online tool (https://henke.lbl.gov/optical_constants/).

Element	Excitation energy [keV] (absorption length [µm])	Kα fluorescence line [keV] (absorption length [µm])
Ca	4.04 (5)	3.69 (18)
P	2.14 (5)	2.01 (5)
Mg	1.3 (1.5)	1.25 (1.5)

S1.2. Matrix composition of different HAB polymorphs

The XRF fitting procedure requires knowledge about the matrix composition, which is used to calculate the absorption of the incoming and fluorescent photons in the sample. Aim of this study is to analyse the content of the MC, and not of the surrounding inorganic tissue. Here we adopt the theoretical composition of HAP-Type B as approximation for the matrix composition: $Ca_9(PO_4)_5(CO_3)_1(OH)_1$ as input to the fitting model.

Table S2 Theoretical and experimental stoichiometry of different HAP polymorphs. It should be noted that the B-type can contain additional HPO_4 ions.

cHA	Experimental	$Ca_{9.7}(PO_4)_{5.9}(CO_3)_{0.2}(OH)_{1.6}$

cHA	Theoretical	Ca10$(PO_4)_6(CO_3)_{0.2}(OH)_{1.6}$
cHB	Experimental	$Ca_{8.9}(PO_4)_{5.3}(CO_3)_{1.1}(OH)_{0.9}$
cHB	Theoretical	$Ca_9(PO_4)_5(CO_3)_1(OH)_1$

Here we use the composition of HAP type B for the matrix with the following mass fractions for the calculations in PyMCA: Ca: 0.39563, P: 0.1697, O: 0.42077, C: 0.013161, H:0.001103.

S1.3. Calculation of effective sample thickness

For the XRF measurement of the thin sections, both fluorescence and transmission data were taken for each pixel. The sample transmission T is defined by $T=I/I_0$, where I is the transmitted intensity and I_0, the intensity of the incoming beam. Both I and I_0 were measured on the same diode, about 15 cm downstream of the sample. The value for I_0 was taken from the transmission through a region without any biological sample, but through the carrier foil. Using the stoichiometry of the ideal HAP type B crystal, the transmission T was calculated using the Henke tables (Henke *et al.*, 1993) via the online tool: (https://henke.lbl.gov/optical_constants/filter2.html) resulting in T_{42}=0.12544 for 4.2 keV, T_{25}=0.11835 for 2.5 keV and T_{21} = 0.15745 for 2.1 keV for a sample of 10 µm thickness d_{10}. The absorption coefficient μ can then be calculated by μ=- ln(T)/d_{10}. Using the measured transmission in each pixel, the effective thickness d_{exp} can be estimated using the absorption coefficient μ by d_{exp}=-ln(T)/μ. This estimate assumes that the sample matrix is HAP type B, which is a good approximation in the region of the MC, but not outside of the MC. In this region, the derived value for the effective thickness has no physical meaning. In this region the estimate for the effective thickness d is direct measure of the X-ray absorption (i.e. the optical density OD) outside of the sample, reflecting the structure outside of the MC itself. Estimated images for the effective thickness derived at different energies are shown in Figure S1. The consistency of the images underlines the validity of the approach.

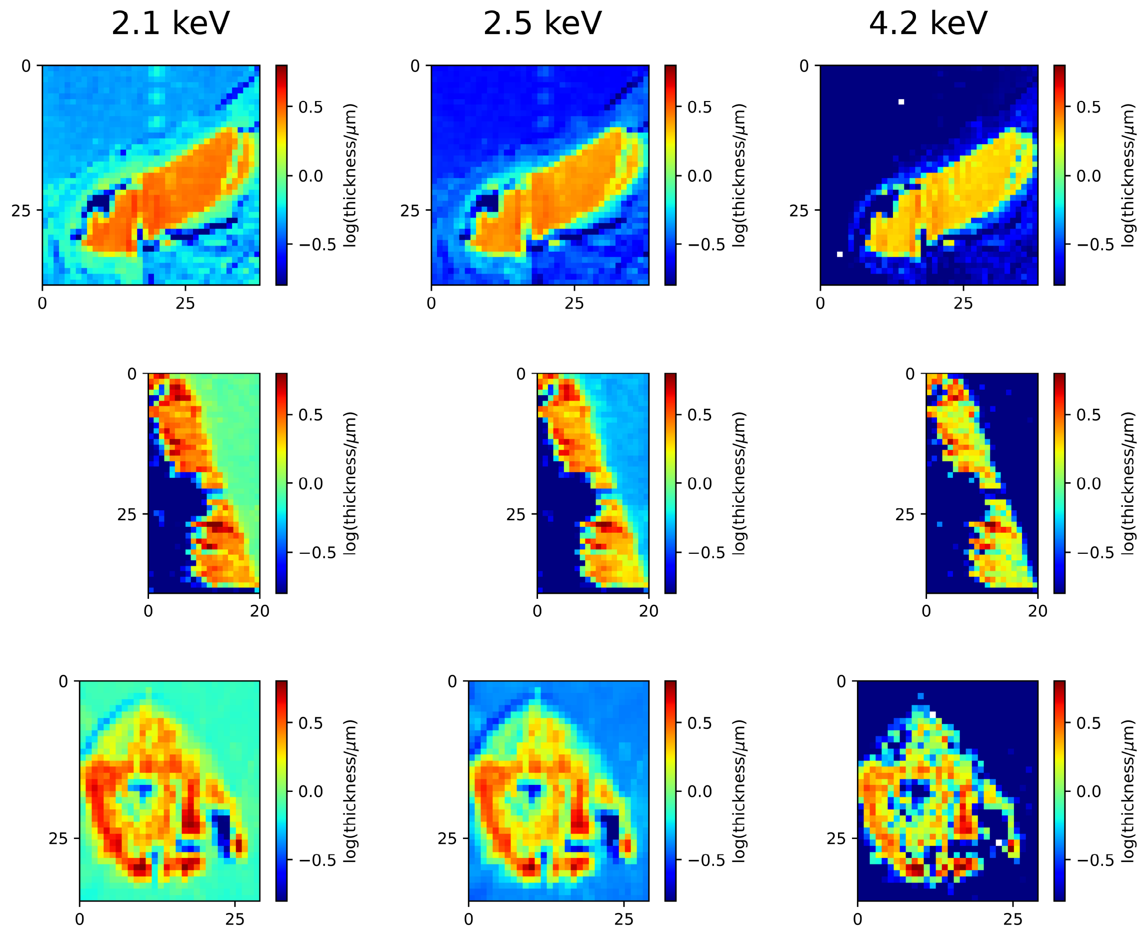

Figure S1 Thickness estimates for all three sample as derived from the x -ray absorption at different energies.

S1.4. Impact of foil contaminations

The samples were mounted on a mylar foil (composition $C_{10}H_8O_4$). Due to the production process of the foil, it contains a small amount of calcium phosphate crystals, which is smaller than the amount present in the MC samples studied here. In Figure S2 we compare the averaged XRF spectra of a MC free region (‘I_0’) of the mylar foil with one derived from the images shown in this paper. In both cases all pixels of the image of the MC and its surrounding and of the region used for the I_0 measurement were averaged. Note that the average sample image contains signal from both the MC and the surrounding region. Obviously, the foil contains several elements, most notably O, Si, Al, and Cl and, as expected, a small amount of P and Ca. For Na and Mg peaks are barely detectable and small compared to the signal from the MC.

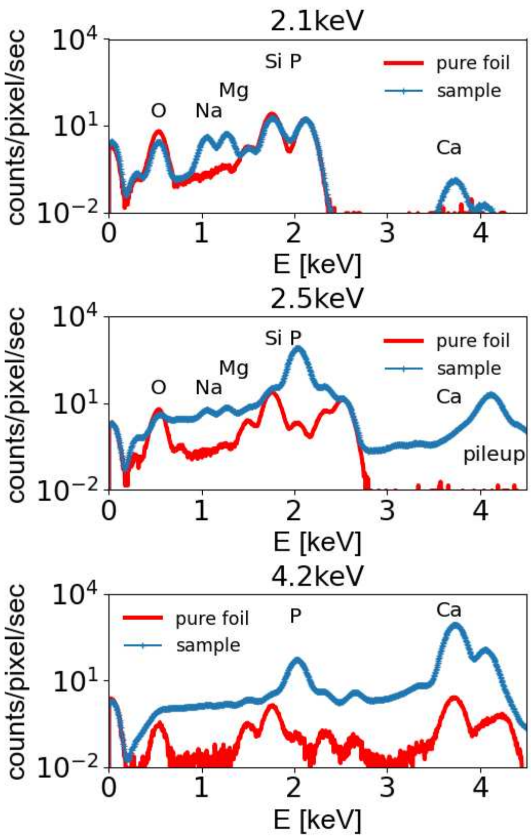

Figure S2 Average XRF spectra from an image used for I_0 calculation (red) and from image taken from the MC and surroundings (blue). Note that the average contains all image pixels, including MC region and regions free of MC. Data are shown in units of counts per pixel/second.

To quantify the amount of contamination, the averaged spectra have been fitted using PyMCA using the same parameters as used when fitting the image pixelwise. Table S3 summarizes the fitted peak intensity for the different elements for all three samples and three excitation energies. The column 'sample' refers to the average signal from the MC and its surroundings, the column 'I_0' to a region with Mylar foil only. Peak intensities are shown in units of counts per seconds per pixel and compared directly with the fitted signal presented in the elemental image. Therefore, the data shown in the column 'I_0' are an estimate of the contribution of contaminations in the foil to the overall signal. We refrained from subtracting this background from the overall signal because it is mostly negligible for the data interpretation.

The column 'I_0/Sample' presents the relative contribution of contaminations to the signal from the MC and surroundings. From Table S3, Table S4, and Table S5 it is clear that the signal from Si and Al is hampered by contaminations, while background contribution for P and Ca measured at 2.5 and 4.2 keV are always below 0.5% and can be ignored. Values for the background are: P(2.5 keV): 30-50 cps/pixel; P(4.2 keV): 2-3 cps/pixel; Ca(4.2 keV) 70-90 cps/pixel. At 2.5keV, the background for Mg

is in the order of 0.3-2.3 cps/pixel, for Na about 0.5-1 cps/pixel corresponding to a contribution less than a few percent.

Table S3 Benign sample: Average count rate for the MC and surroundings and I_0 at different excitation energies. Columns 'sample' and 'I_0' refer to the average signal in the MC and surroundings and a region with foil only that supports the sample taken for I_0 respectively.

Element	Sample [cps/pixel]	I_0 [cps/pixel]	I_0/Sample
2.1keV			
O K	55.71	164.48	2.95
F K	1.41	0.07	0.0506
Na K	68.36	0.04	0.000529
Mg K	181.31	0.05	0.000267
Al K	34.86	44.29	1.27
Si K	569.56	814.16	1.43
2.5 keV			
Na K	40.24	0.49	1.21
Mg K	109.35	0.31	0.00283
Al K	13.83	51.84	3.75
Si K	695.54	657.29	0.945
P K	19200.58	39.41	0.00205
S K	1731.07	17.51	0.0101
Fe L	9.72	2.66	0.2746
4.2 keV			
Na K	2.43	0.12	4.94
Mg K	4.76	0.24	0.0501
Al K	9.97	6.19	0.621
Si K	39.43	31.61	0.802
P K	963.31	2.19	0.00227
S K	61.98	0.44	0.00714
Cl K	47.51	41.13	0.865

K K	38.68	0.37	0.00955
Ca K	21034.26	95.45	0.00454
Fe L	2.85	0.01	0.00421

Table S4 IDC Sample: Average count rate for MC and surroundings and I_0 at different excitation energies. Columns 'Sample' and 'I_0' refer to the average signal in the MC and surroundings and a region with only the foil that supports the sample taken for I_0 respectively.

Element	Sample [cps/pixel]	I_0 [cps/pixel]	I_0/Sample
2.1 keV			
O K	68.68	152.27	2.22
F K	1.64	0.37	0.225
Na K	102.73	0.66	0.00646
Mg K	128.38	2.94	0.0229
Al K	26.41	45.02	1.71
Si K	576.71	765.88	1.33
2.5 keV			
Na K	62.69	0.94	0.0149
Mg K	72.32	2.31	0.0319
Al K	24.03	57.25	2.38
Si K	800.65	697.20	0.871
P K	25542.76	45.59	0.00179
S K	890.52	153.04	0.172
Fe L	4.34	0.26	0.0610
4.2 keV			
Na K	5.53	0.27	0.0481
Mg K	4.20	0.31	0.0736
Al K	17.47	8.40	0.481
Si K	56.08	38.03	0.678
P K	1443.79	2.17	0.00150
S K	34.15	3.68	0.108
Cl K	70.44	2.11	0.0299
K K	89.54	0.74	0.00826
Ca K	34466.17	98.01	0.00284

Fe L	6.08	0.10	0.0170

Table S5 Sample DCIS: Average count rate for MC and surroundings and I_0 at different excitation energies. Columns 'Sample' and 'I_0' refer to the average signal in the MC and surroundings and a region with only the foil that supports the sample taken for I_0 respectively.

Element	Sample [cps/pixel]	I_0 [cps/pixel]	I_0/Sample
2.1 keV			
O K	104.63	151.96	1.45
F K	3.59	0.10	0.0290
Na K	139.15	0.87	0.00628
Mg K	398.69	0.73	0.00182
Al K	27.93	46.56	1.67
Si K	1596.36	852.98	0.534
O K	104.63	151.96	1.45
2.5 keV			
Na K	84.33	1.09	0.0129
Mg K	251.85	0.86	0.00341
Al K	30.42	59.83	1.97
Si K	1779.44	737.75	0.415
P K	38796.14	44.30	0.00114
S K	1075.89	11.55	0.010735
Fe L	1.73	0.20	0.117
4.2 keV			
Na K	6.33	0.11	0.0181
Mg K	11.51	0.29	0.0254
Al K	21.99	8.14	0.3697
Si K	109.80	42.86	0.390
P K	2127.68	2.44	0.001146
S K	37.28	0.40	0.0107
Cl K	101.94	2.25	0.0221
K K	64.23	0.48	0.00742

Ca K	51736.51	75.29	0.00146
Fe L	8.7	0.0	0.00636

S1.5. Potential pitfalls of quantitative data analysis

The presented quantitative analysis of the XRF data relies on the assumed geometry and the physical data used for the simulation of the XRF spectra as implemented in PyMCA. The data were taken under grazing exit conditions with the detector mounted under 90° relative to the incoming beam, and the fluorescent light leaving the sample under an angle of 6°. Sample geometry and thickness are critical parameters for the quantitative analysis because the absorption length of the incoming radiation (2.5 keV ~6 µm, 4.2 keV: 9 µm in HAP) differs from the absorption length of the fluorescent light from the sample. While the model implemented in PyMCA considers these effects, systematic errors can occur due to deviations between ideal and real geometry, most notably due to 1) the finite detector opening 2) uncertainties in determination of the scattering angle and 3) local tilts of the not perfectly smooth sample surface.

S1.5.1. Impact of geometry errors on estimated spatial distribution of P and Mg

The observed effective thickness variations in the MC may indicate that the sample either has a rough surface, or that the sample matrix is non-homogeneous. In principle, the angle between surface and incoming beam could vary from 0 to 90 degree. With a beamsize of about 22 µm and the average sample thickness of about 5 µm, the average, effective tilt angle of the sample surface relative to the ideal geometry cannot exceed 15-20 degree. Even if there are local regions with a larger tilt angle in the illuminated surface region, there will always be a region in the beam which is tilted in the opposite direction, at least partially averaging out artefacts. While quantification is beyond the scope of this paper, we try to assess the magnitude of such effects by evaluation of the data using different geometric assumptions. Figure S3 shows the calculated P and Mg mass fraction as derived from data taken at 4.2 keV (for P, left two columns) and 2.5 keV (for Mg, right two columns). The geometry is denoted by the exit angle, which is the angle of the sample surface relative to the detector. The angle between detector and incoming beam is kept constant at 90 degrees. Rows 1-4 refer to the data analysis of the same data set with exit angles of 6, 8, 20 and 45 of the fluorescent photons relative to the sample surface, respectively. The plots in columns 2 and 4 show the estimated mass fraction of P and Mg as function of the effective sample thickness, plotted for each pixel. The red line in the plots for w_P marks the theoretical value of 0.169 for P in HAP type B. For an exit angle of 6-8 degree, and for an effective thickness of 2-3 µm (i.e. within the MC), the calculated value for w_P coincides well with the value derived from the data, providing confidence in the overall data analysis (Figure S3 row 1 and 2, second column). While the absolute estimate for the mass fraction depends on the assumed sample geometry analysis (compare plots in column 2 of Figure S3), the spatial distribution of the estimated mass fraction within one sample remains unchanged, when using a ‘wrong’ sample geometry, as illustrated in column 1 and 3. This confirms that the features of the ***derived spatial elemental distribution,*** for both P and Mg are robust against errors in geometric assumptions.

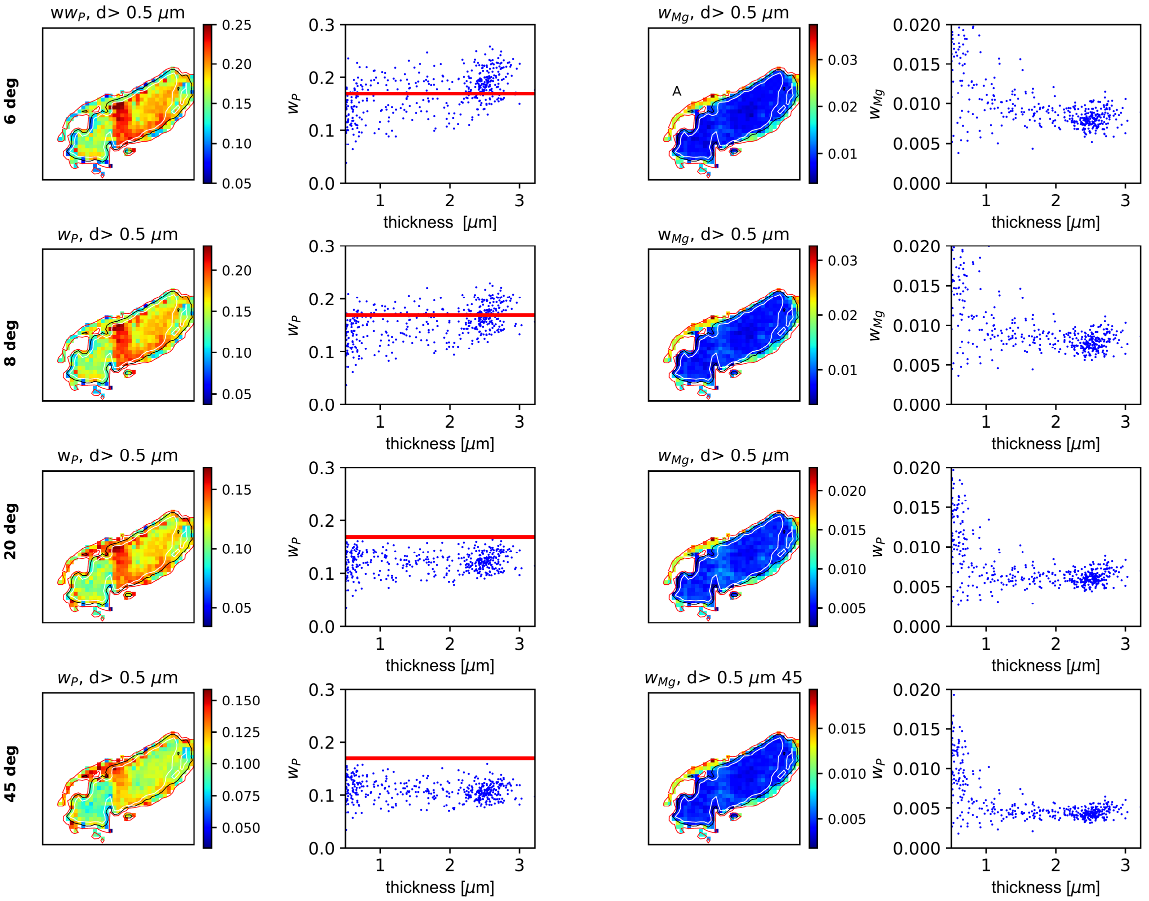

Figure S3 Simulated estimates for the mass fraction of P (column 1 and 2) and Mg (columns 2 and 3) assuming different exit angles of 6, 8, 10, and 45 degrees using the same raw data set. Columns 2 and 3 show the estimated mass fraction for each pixel, as derived from the fit to the XRF spectra in each pixel.

S1.5.2. Impact of geometry errors on mass fraction as function of sample thickness.

Estimating the mass fraction as average value for certain size bins provides additional insight. As described in the main text, here we fit the PyMCA model to an average spectrum for each size bin. Comparing Figure S4b,c (for 20 degree) and Figure S5b,c (for 45 degree) with Figure 6 shows that the estimate for the total Mg mass fraction is sensitive to changes of the assumed geometry. However, all model calculations show enhanced Mg in the thinner MC regions only for the benign sample, and the lowest Mg concentration is found in the IDC sample, even if an unphysical scattering angle of 45° is used. Consequently, we can conclude with a high degree of confidence that the observed enhancement of the Mg mixing ratio on the MC edges of the benign sample when compared to the other samples is not a geometrical artifact.

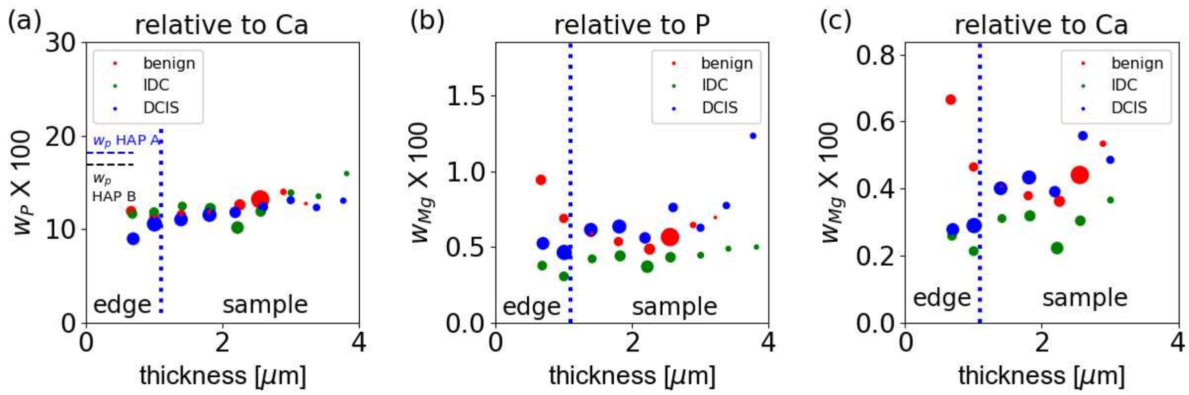

Figure S4 Estimated mass fraction as function of effective sample thickness, assuming an exit angle of 20 degree of the sample surface relative to the detector. (a) P mass fraction as derived from data taken at 4.2 keV, (b) Mg mass fraction derived from data taken at 2.5keV, assuming that P has the theoretical mass fraction of 0.196. (c) Mg mass fraction, derived from data at 2.5 keV and 4.2 keV, assuming that Ca has the theoretical mass fraction of 0.39. The chosen size bins for the effective thickness are: 0.6-0.8, 0.8- 1.2, 1.2-1.6, 1.6-2.0, 2.0-2.4, 2.4-2.8, 2.8-3.2, 3.2-3.5, 3.6-4.0 µm. The size of the points is proportional to the number of spectra averaged.

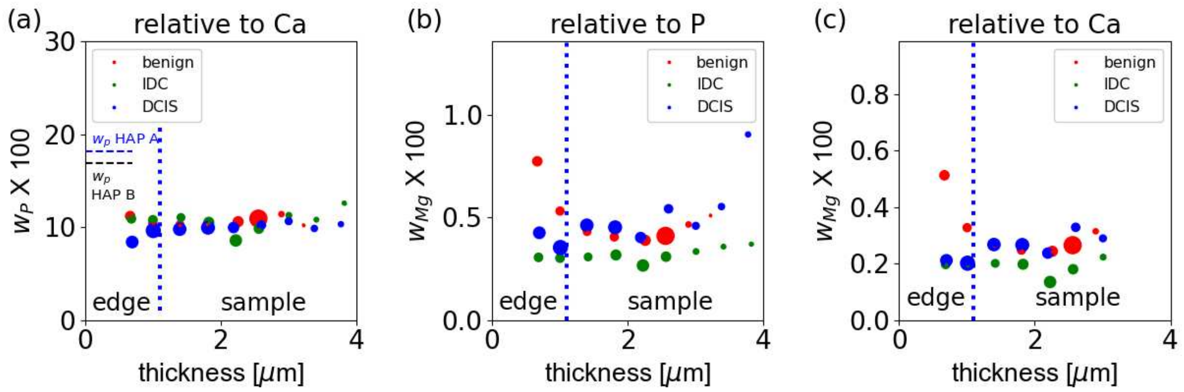

Figure S5 Estimated mass fraction as function of sample thickness, assuming an exit angle of 45 degree of the sample surface relative to the detector. See also Figure S4.

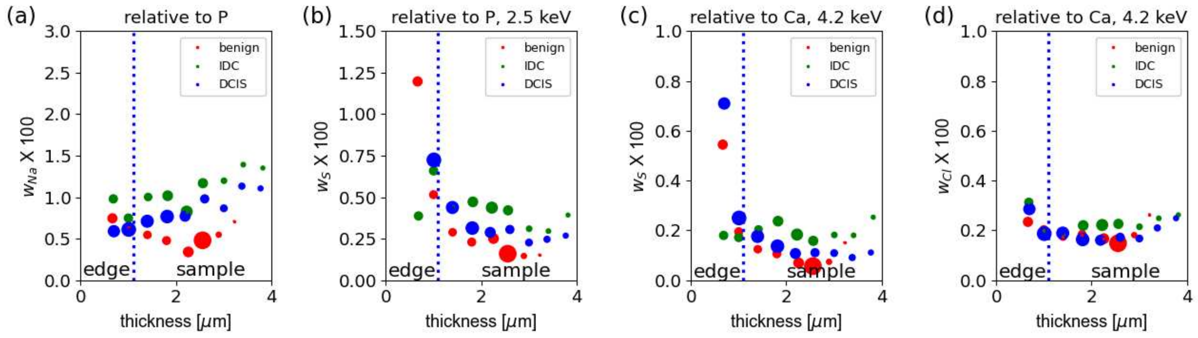

Figure S6 Estimated mass fraction as function of effective sample thickness assuming a scattering angle of 20 degree. (a) Na mass fraction as derived from data taken at 2.5 keV, (b) S mass fraction derived from data taken as 2.5keV, assuming that P has the theoretical mass fraction of 0.196. (c) S mass fraction, derived from data at 4.2 keV, assuming that Ca has the theoretical mass fraction of 0.39. See also Figure S4.

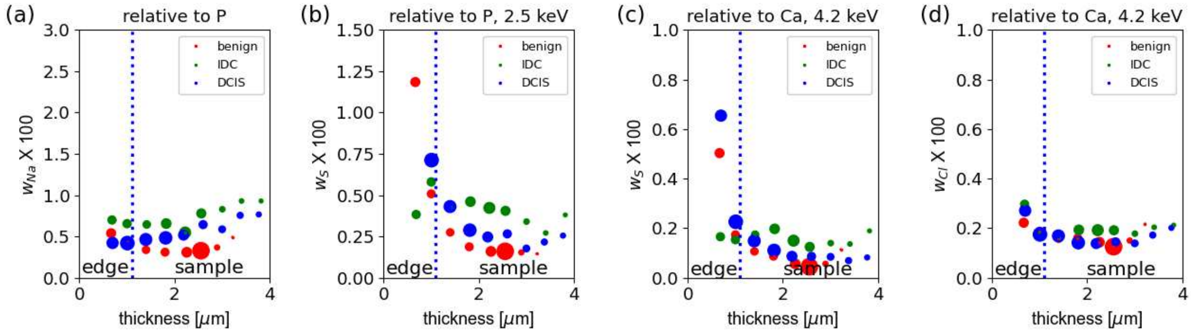

Figure S7 Estimated mass fraction as function of effective sample thickness, assuming an exit angle of 45 degree of the sample surface relative to the detector. See also Figure S4.

S1.6. Fluorescence of Si, Sr and Y

Figure S8 compares different fitting strategies for XRF spectra taken at 2.1 keV. While the Kα emission lines of the light elements Na, Mg, and Al can be fitted nicely to the data, the region between the Kα emission line of Si and the scattering line remains unexplained, as indicated by the red arrow in Figure S8 (a) and (b). Furthermore, when recording the spectrum using an energy dispersive detector with about 150 eV energy resolution, the candidate fluorescence peaks of Si ($K\alpha_1$: 1.739 keV, Kβ1: 1.836 keV), Sr ($L\alpha_1$: 1.806 keV, $L\alpha_2$: 1.804 keV and $L\beta_1$: 1.872 keV), Y ($L\alpha_1$: 1.923 keV , $L\alpha_2$: 1.921 keV and $L\beta_1$: 1.996 keV) and P ($K\alpha_1$: 2.013 keV, $K\beta_1$: 2.129 keV) partially overlap and are in the vicinity of the elastic scattering line, which is at the same energy as the energy of incoming photons (2.1V keV).

While the P fluorescence line cannot be excited at photon energies below the P absorption edge (2.145 keV), additional complications can arise from the unavoidable presence of photons from higher harmonics passing the monochromator. Here we use a Si 111 crystal, which blocks the second harmonics, but not the third harmonics (6.3 keV). The high harmonics light is suppressed in the beamline by three reflections (0.4 degree) on a Ni coated mirror. While the presence of the remaining high harmonics (typically well below 0.1 %) can be disregarded in the majority of circumstances, this is not necessarily the case when examining small quantities of trace elements within a matrix of elements, which are to a lesser degree excited by high harmonics. For example, assuming that 1) the

HAP matrix contains 0.1% Sr, 2) equal cross and absorption sections for Sr and P, and 3) that incoming photons of 2.1 keV contain 0.1 % of high harmonics photons at 6.3 keV, then the emission lines of Sr and P would be of equal intensity. As these lines partially overlap, the excitation of P would affect the quantitative determination of Sr. Figure S8 illustrated different fitting strategies. All fits shown in Figure S8, assume 0.1% of high harmonics contamination (photons of 6.3 keV), which allows to fit the Ca Ka peak. As the ratio of the Ca Kα and P Kα emission lines is physically fixed as both lines are only exited by the higher harmonic contribution of 6.3 keV, the intensity P emission line at 2 keV (blue dashed line) is independent of the assumed contamination with high order light. This excludes that there is a significant excitation of the P emission line from the HAP matrix due to high order contamination.

Figure S8 (a-e) shows different fitting strategies. Figure S8 (a) shows that assuming the HAP type B matrix and light elements is not sufficient to fit the red marked region (1.7-2 keV). Although we have excluded any significant excitation of the P emission line, allowing P as fitting parameter to account for deviations from the ideal matrix composition (Figure S8 b), or allowing both Sr and P (Figure S8 c) would not fit the region between 1.7 and 2 keV). Similarly, fixing P to the matrix composition and adding Sr (Figure S8 d), or Sr and Zr (Figure S8 e), will not fit the region 1.7-2 keV. The only viable way it to add Sr and Y to the fit, as shown in Figure S8f. Figure S8g show that the composition of the foil can be fitted nicely, if one allows the HAP type B matrix and light elements. The fit in the region between 1.7-2 keV cannot be improved by allowing P as fitted parameter to the fit. Finally, Figure S8i shows that allowing Y and Sr to the fit of the clean foil has no effect and that the fitted amount of Y is low compared to the amount of Y found in the sample.

Mass fractions have been derived from fitting the XRF model (Figure S8f) to the data for each pixel (See Figure 5). For the Y and Sr mass fraction maps, data from four pixels have been averaged to ensure sufficient data quality to disentangle the overlaying peaks of Sr, Y and Si. This corresponds to a boxcar smoothing of the data in the spatial domain.

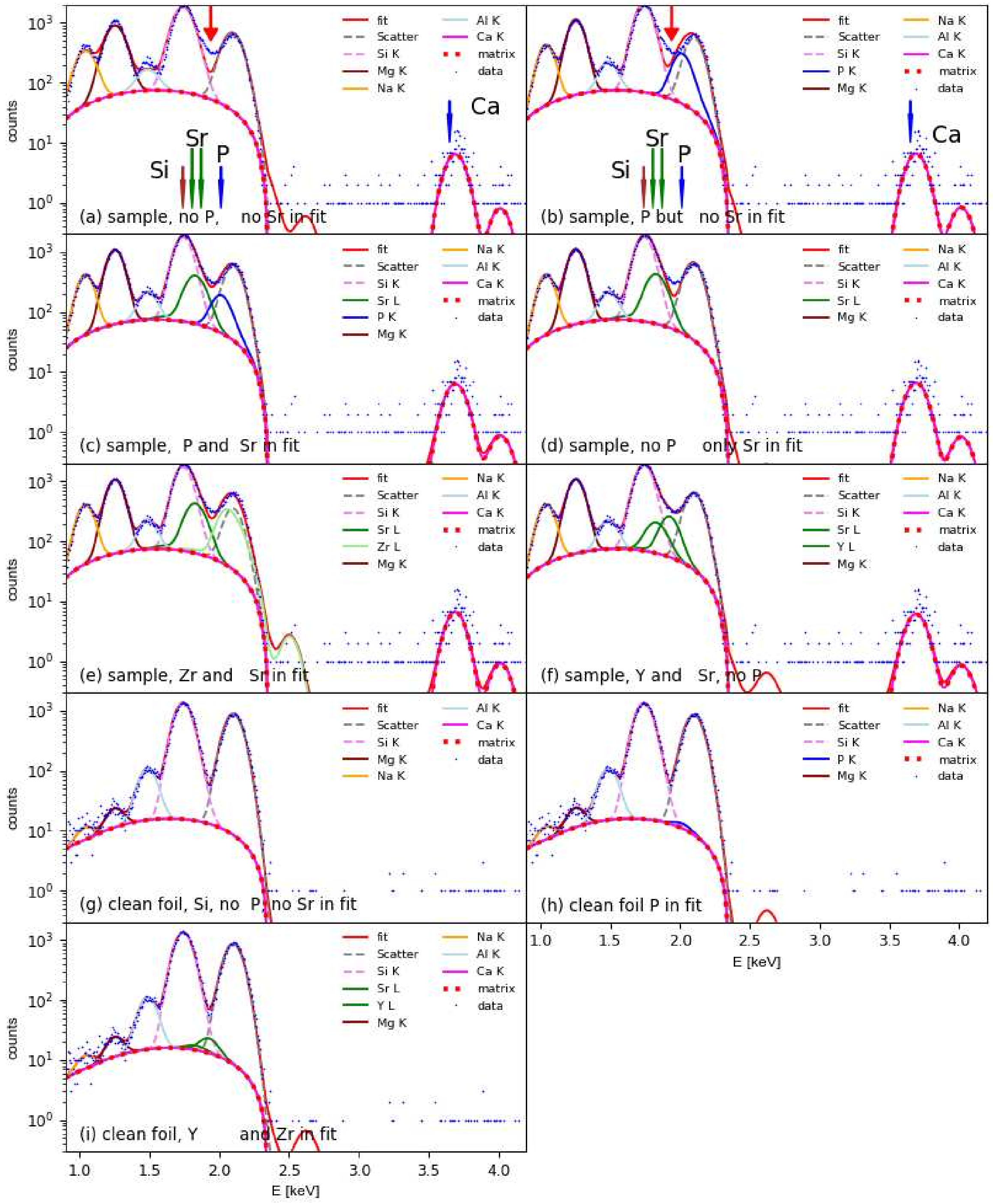

Figure S8 Different fitting strategies to determine the presence of Sr in the sample Data taken from position '*' as marked in Figure 2 of the main text. Nine point spectra have been averaged.

S2. XANES point spectra

The spectra shown in Figure S9 are measured along a vertical (h1-h11) and a horizontal line (v1-v10) shown in Figure8a. The broad peak around 4060 eV that can be seen in h1, h2 and h3 positions indicates the presence of calcite in the region outside MC. The other spectra have similar shapes of the pre-edges and white lines, but the energy position of the white line peaks changes by about 1 eV indicating slight changes in composition, as shown in Table S6 and Table S7.

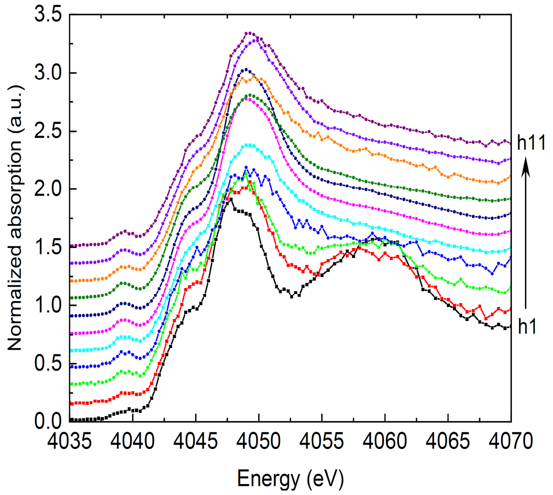

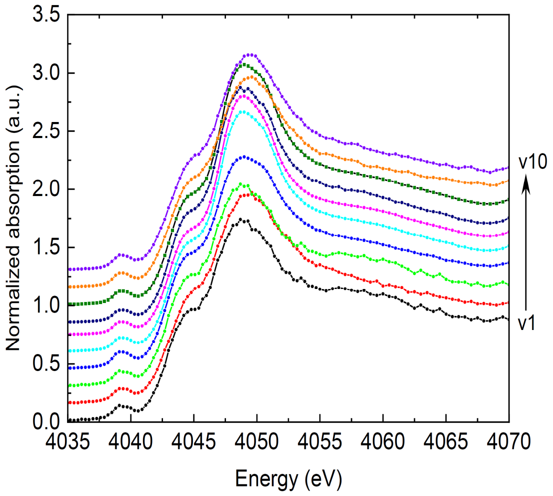

Figure S9 Ca K-edge XAS spectra in all locations as shown on map in Figure 8.

Table S6 Results of the linear combination fitting for the benign sample along the lines marked in Figure 8 using five reference spectra: WHIT, HAP, HAP type A, HAP Type B and Calcite.

Position	WHIT %	HAP %	HAP Type A %	HAP Type B %	Calcite %
h1	0	0	0	2.4	97.6
h2	18.1	9.4	0	10.5	62.5
h3	32.6	0	0	4.7	61.7
h4	47.2	0	0	11.8	37.2
h5	57.6	0	14.2	0	25.1
h6	58.1	10.1	25.5	0	5
h7	31.4	23.5	25	18.2	0
h8	58.7	0	15.9	3.9	15.9
h9	24.1	0	54.2	0	19.3

h10	77.2	0.5	4.9	0	16
h11	29.4	2.2	5.4	0	12.9
v1	44.3	0	14.6	0	37
v2	71.7	0.2	2.8	0	22.1
v3	28	24.7	24.3	23.5	0
v4	3	8.7	68.5	13.4	7.3
v5	35.6	15.8	43	0	5.9
v6	34	18.8	38.8	0	6.1
v7	38.4	18.4	33.5	0	9.4
v8	37.1	13.5	45.2	0	4.8
v9	70.1	0	0	4	22.5
v10	74.7	0	3.8	0	18.4

Table S7 Results of the linear combination fitting for the benign sample along the lines marked in Figure 8 using six reference spectra: WHIT, HAP, HAP type A, HAP Type B, Calcite and ACC.

Position	*WHIT* %	*HAP* %	*HAP_A* %	*HAP_B* %	*Calcite* %	*ACC* %
h1	0	0	7.5	0	95.9	4.1
h2	0	0	7.9	3.2	59.2	31.3
h3	7.2	0	0	0	56.5	36.2
h4	38.8	0	0	0	33.3	24.9
h5	30.6	0	0	0	16.4	50.1
h6	40.8	0	34.5	0	1.7	22.3
h7	34.5	48.7	10.2	0	0	7.6
h8	60.1	0	0	0	13.2	20.8
h9	18.4	0	0	0	17.8	61.6
h10	60.2	0	0	0	6	32
h11	32.8	0	0	0	10.3	55.4
v1	37	0	0	0	34.3	24.9

v2	40.5	0	0	0	12.8	40.5
v3	55.7	0	42.4	0	0	4.1
v4	32.2	8.2	0	0	0	58.1
v5	29.4	51.6	0	0	0	21.2
v6	22.8	0	60.3	0	0	16.7
v7	24.8	43.6	0	0	3.5	29.7
v8	34.9	47.5	0	0	0	19.9
v9	32.2	0	0	0	17	50.8
v10	59.2	0	0	0	14.5	23.6

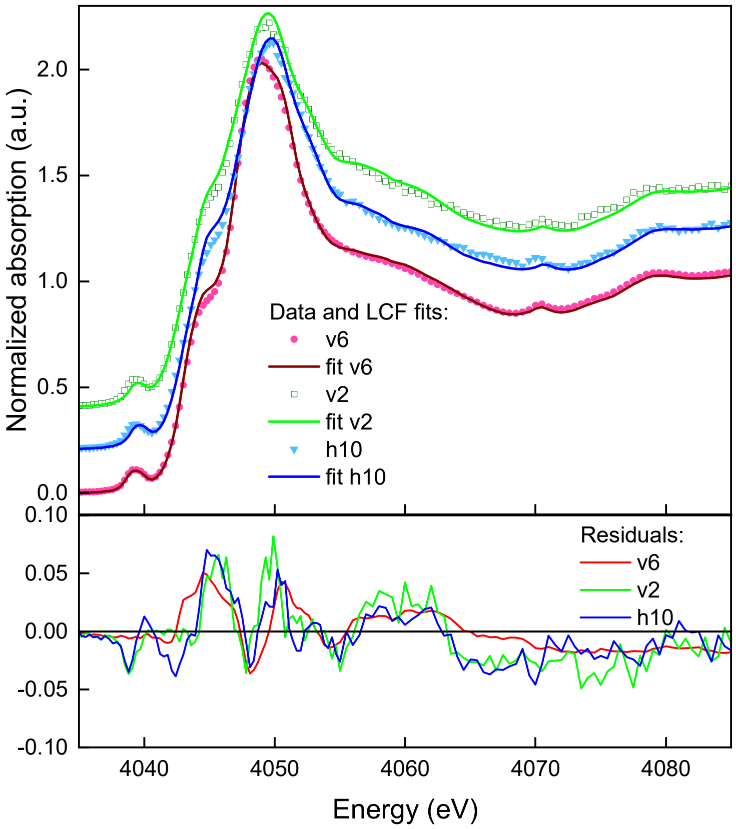

Figure S10 Measured spectra and the LCF fits in three representative points: v6 in the centre, v2 and h10 on the edges. The fit residuals are plotted separately, at the bottom.

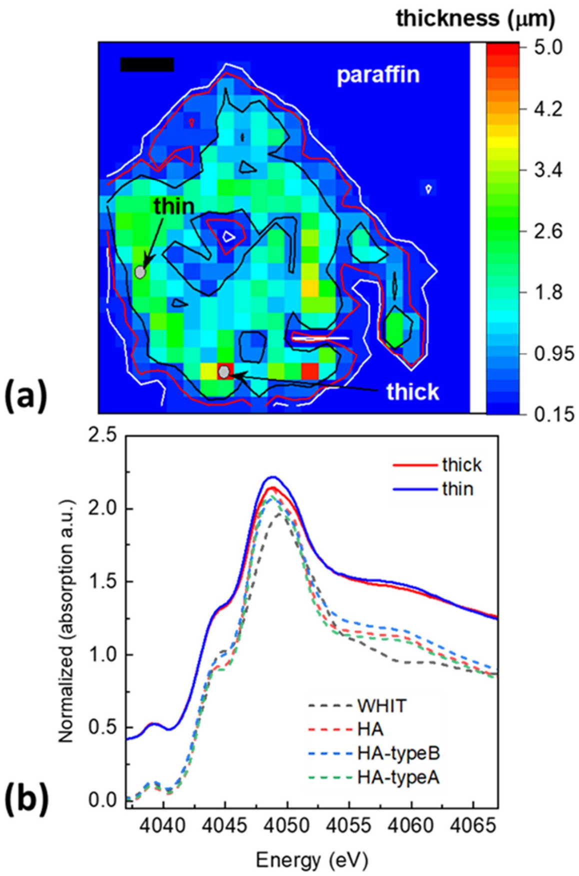

Figure S11 (a) Effective thickness of the DCIS sample recorded at 4200 eV incident energy (scale bar is 100 microns). Three contour lines delineate the effective sample thickness at 0.3 (white), 0.5 (red) and 1 micron (black) calculated using HA density. (b) Ca K-edge XAS spectra for two points (continuous lines) representing thick and thin regions, as well as for different phosphate containing references (dotted lines).

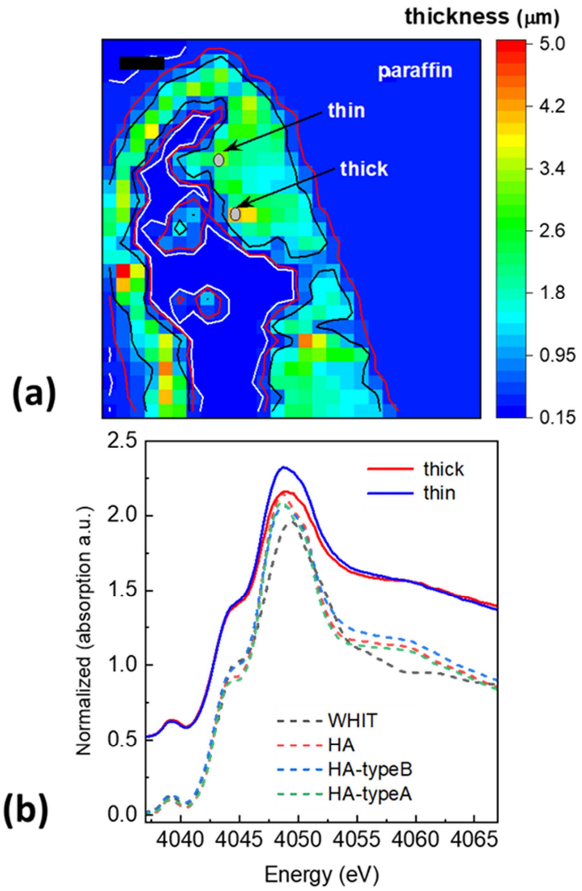

Figure S12 (a) Effective thickness of IDC sample recorded at 4200 eV incident energy (scale bar 100 microns). Three contour lines delineate the effective sample thickness at 0.3 (white), 0.5 (red) and 1 micron (black) calculated using HA density. (b) Ca K-edge XAS spectra for two points (continuous lines) representing thick and thin regions, as well as for different phosphate containing references (dotted lines).

Table S8 LCF fits for DCIS and IDC samples shows the HA type B as a major mineral component.

	WHIT %	HA %	HA Type A %	HA Type B %	Calcite %
DCIS_thick	0	4.2	5.3	79.9	0
DCIS_thin	0	0	4.6	86.3	0
IDC_thick	0	6.7	6.7	77.7	0
IDC_thin	0	3.5	3.6	85	0
